# Assessing petroleum contamination in parts of the Niger Delta based on a sub-catchment delineated field assessment

**DOI:** 10.1007/s10661-024-12743-7

**Published:** 2024-05-29

**Authors:** Ibukun Ola, Carsten Drebenstedt, Robert M. Burgess, Martin Mensah, Nils Hoth, Precious Okoroafor, Christoph Külls

**Affiliations:** 1 Institute of Mining and Special Civil Engineering, Technical University Mining Academy Freiberg DE, Gustav-Zeuner Street 1A, 09599 Freiberg, Germany; 2 U.S. Environmental Protection Agency, Office of Research and Development, Center for Environmental Measurement and Modeling, Atlantic Coastal Environmental Sciences Division, 27 Tarzwell Drive, Narragansett, Rhode Island 02882 USA; 3 Institute of Biosciences/Interdisciplinary Environmental Research Centre, Freiberg Technical University of Mining, Leipziger Street 29, 09599 Freiberg, Germany; 4 Labor Für Hydrologie Und Internationale Wasserwirtschaft, Technische Hochschule, 23562 Lübeck, Schleswig-Holstein Germany

**Keywords:** Niger Delta, Petroleum contamination, Sub-catchment assessment, Environmental remediation, Hydrological analysis

## Abstract

The Niger Delta in Nigeria is a complex and heavily contaminated area with over 150,000 interconnected contaminated sites. This intricate issue is compounded by the region’s strong hydrological processes and high-energy environment, necessitating a science-based approach for effective contamination assessment and management. This study introduces the concept of sub-catchment contamination assessment and management, providing an overarching perspective rather than addressing each site individually. A description of the sub-catchment delineation process using the digital elevation model data from an impacted area within the Delta is provided. Additionally, the contamination status from the delineated sub-catchment is reported. Sediment, surface water and groundwater samples from the sub-catchment were analyzed for total petroleum hydrocarbons (TPH) and polycyclic aromatic hydrocarbons (PAHs), respectively. Surface sediment TPH concentrations ranged from 129 to 20,600 mg/kg, with subsurface (2-m depth) concentrations from 15.5 to 729 mg/kg. PAHs in surface and subsurface sediment reached 9.55 mg/kg and 0.46 mg/kg, respectively. Surface water exhibited TPH concentrations from 10 to 620 mg/L, while PAHs ranged from below detection limits to 1 mg/L. Groundwater TPH concentrations spanned 3 to 473 mg/L, with total PAHs varying from below detection limits to 0.28 mg/L. These elevated TPH and PAH levels indicate extensive petroleum contamination in the investigated sediment and water environment. Along with severe impacts on large areas of mangroves and wetlands, comparison of TPH and PAH concentrations with sediment and water quality criteria found 54 to 100% of stations demonstrated exceedances, suggesting adverse biological effects on aquatic and sediment biota are likely occurring.

**Supplementary Information:**

The online version contains supplementary material available at 10.1007/s10661-024-12743-7.

## Introduction

The Niger Delta region of Nigeria is a vast area spanning nine states in the southern part of the country, known for its rich wetland ecosystem and dense population (Adekola & Mitchell, 2011; Nwilo and Badejo, 2005; Kadafa, 2012). It is home to diverse flora and fauna, including rare species, and serves as critical habitat (Adekola & Mitchell, 2011; Allison et al., 2018; Ugochukwu & Ertel, 2008). The region holds significant ecological and economic importance due to its abundant natural resources, including petroleum, gas and timber, which contribute to Nigeria’s national revenue. Fishing is also a vital economic activity, with many communities relying on the wetlands for their sustenance and livelihoods.

However, the exploration and production of oil in the Niger Delta have had a severe impact on its environment. Large-scale water and air pollution, land degradation and deforestation have resulted from oil spills (Jernelöv, 2010; Lindén & Pålsson, 2013; Pegg & Zabbey, 2013). The landscape of the Delta is dotted with pipelines and pumping stations, often in close proximity to local communities, leading to contamination of farmlands, fisheries and drinking water sources (Jernelöv, 2010). These spills have caused food and water insecurity among the already vulnerable rural communities.

Oil spills in the Niger Delta can be attributed to both operational factors, such as aged and poorly maintained pipelines, and non-operational factors, including vandalism, oil theft and sabotage. There remains some uncertainty about the differences in the magnitude of operational spills versus non-operational spills (Babatunde, 2020; Bodo, 2019; Boele et al., 2001).

Two methods are currently employed to assess oil spill impacts in the Niger Delta: the traditional assessment and the Shoreline Cleanup Assessment Technique (SCAT). The traditional method involves sample collection and analysis within predefined grids, providing detailed information on contaminant concentrations. Environmental studies (Abbas & Brack, 2006; Gundlach et al., 2021; Lindén & Pålsson, 2013; Olajire et al., 2005) in the Niger Delta have commonly utilized this approach. On the other hand, the SCAT approach involves visually observing the oiling levels within predefined grids and using that information to plan and implement cleanup efforts. A key advantage here is, this approach allows for a participatory approach, where various stakeholders (including the responsible party, regulators, representatives from the local community and NGOs) all view the site at the same time and make a consensus recommendation regarding the appropriate response action. Relatively few studies (Bonte et al., 2020; Little et al., 2018) have used the SCAT approach to investigate oil impacts and to define the wider scope and need for remediation in the Niger Delta. While both methods serve as rapid screening tools, they have limitations. They do not fully consider critical physical parameters such as waves, tides and interconnected hydrological systems, which strongly influence petroleum transport in the Delta (Lindén & Pålsson, 2013). Additionally, they focus on spill area-specific assessments and may not adequately address the complex, extensive and compounding nature of oil spills in the entire region. The interconnected ecosystems and hydrological processes in the Delta necessitate a broader perspective to reflect the regional scale of contamination. Hence, a more comprehensive approach is needed to inform remedial action plans and restoration efforts particularly in the Delta’s complex near shore environment.

Based on that background, it is clear a new method for investigation and assessment is needed, an approach that does not oversimplify the system but rather captures fundamental system dynamics and allows for an adequate assessment of petroleum-related risks. Consequently, this study aims to introduce and elucidate the concept of sub-catchment site assessment and management for petroleum contamination in the Niger Delta. The approach distinguishes itself by using Geographic Information System (GIS) for sub-catchment delineation, which provides a more comprehensive understanding of contamination dynamics and offers a broader framework for assessment and management. In the subsequent sections, this study provides a detailed description and framework for this innovative approach, which offers a holistic view of contamination dynamics within the region’s complex hydrological landscape. Subsequently, the study presents a preliminary investigative assessment of petroleum contamination status within the delineated sub-catchment, focusing on samples of surface and subsurface sediment, groundwater and surface water collected from up to 15 stations along the Kporghor River. The choice of the example sub-catchment is in part because it represents one of the sites earmarked for remediation by Nigeria’s federal environmental agency, Hydrocarbon Pollution Remediation Project (HYPREP). HYPREP is tasked with the responsibility of addressing petroleum-contaminated sites in the Niger Delta. Additionally, security challenges are prevalent in the Niger Delta. Nevertheless, at the study site, robust support from the local community facilitated unobstructed access. Oftentimes, environmental sampling efforts in the region are hindered by community hostility and security concerns (Lindén & Pålsson, 2013).

## Materials and methods

### Study area description

**Fig. 1:**
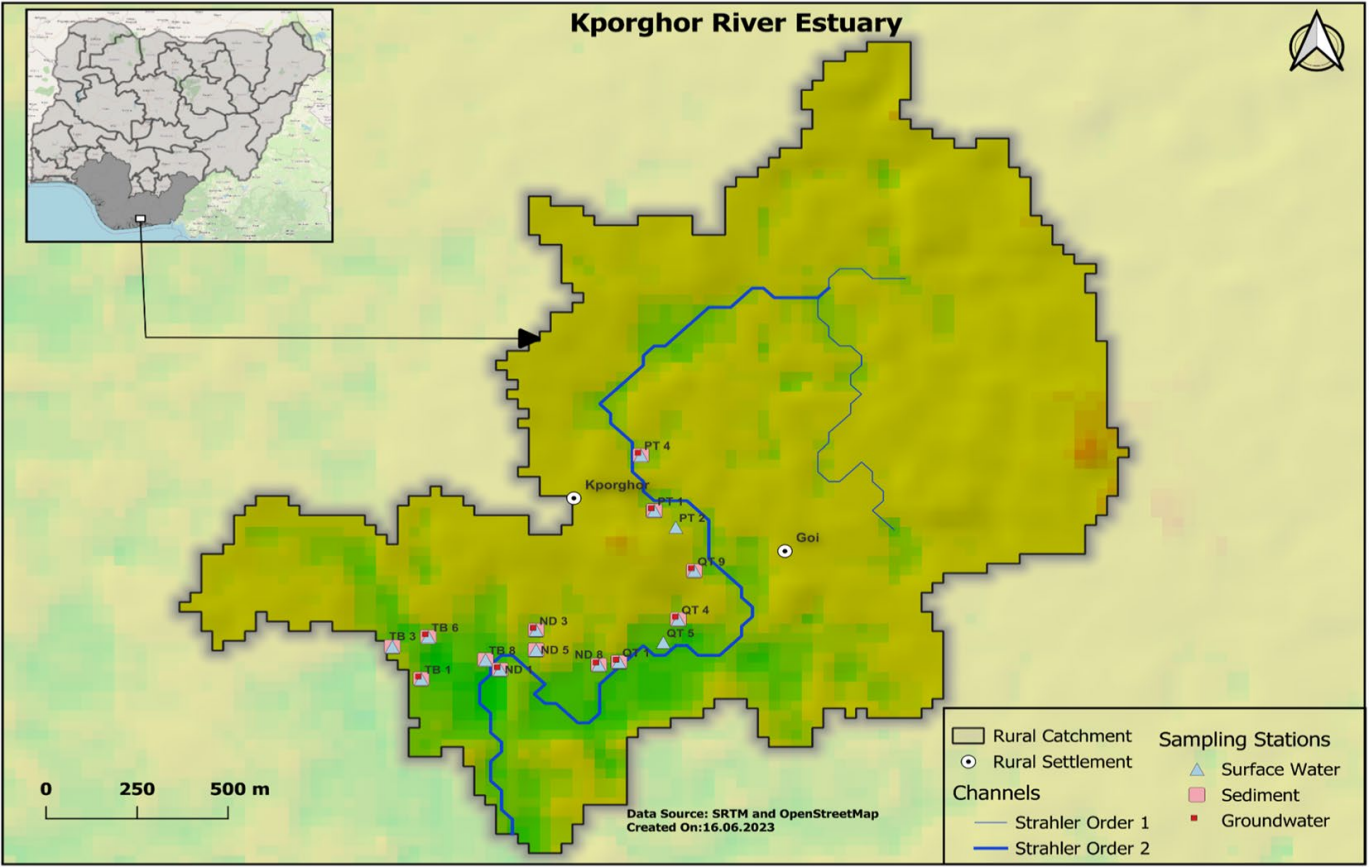
Digital elevation model derived sub-catchment. Data derived from Shuttle Radar Topography Mission (SRTM) 1 Arc-Second global dataset and OpenStreetMap. Sub-catchments shown along with types of samples collected (e.g. sediment, surface water, ground water)

The Kporghor River Estuary sub-catchment is defined by its diverse geographical and environmental features. Geographically, this sub-catchment is located approximately 30 km southeast of the city of Port-Harcourt. It is framed by a gentle topographic relief, with elevations ranging from sea level to approximately 21 m, and is characterized by its generally mild slopes, typically falling within the range of 0–14% (Fig. 1).

The sub-catchment area includes rural settlements, prominently Kporghor and Gio, which contribute to its land use characteristics. Food crop cultivation dominates the landscape, with crops such as maize (Zea mays) and cassava (Manihot esculenta) being prominent. Additionally, forested areas, mainly comprised of diverse rainforest species and mangrove vegetation, are integral to the study area’s environmental composition. The mangrove ecosystem is further influenced by invasive species, notably the Nypa palm (Nypa fruticans, Wurmb). The presence of creeks, wetlands and remnants of large areas of impacted mangrove vegetation underscores the ecological significance of this region.

The climate within the Niger Delta region, including that of the study area, exhibits distinctive seasonal patterns. The region experiences a protracted rainy season extending from March to October, followed by a shorter dry season spanning December to February. The annual rainfall in this region exceeds 3000 mm, as documented by (Adejuwon, 2012). Moreover, evapotranspiration rates in southern Nigeria, covering the Niger Delta, have been computed in previous studies (Ayoade, 1975; Chapas & Rees, 1964; Hayward & Oguntoyinbo, 1987), estimating an annual evaporation of around 1000 mm. Despite frequent cloud cover and limited sunshine hours, mean annual temperatures consistently range from 24 to 32 °C (Osuji & Opiah, 2007).

### Sub-catchment delineation process

The delineation of catchments and sub-catchments involves several key steps. In the following section, those steps are outlined and the relevant scientific literature used in this investigation is cited. The process utilized open source QGIS 3.4.8 and followed the methodology outlined by (van der Kwast & Menke, 2022). The initial step involved data preparation. A Shuttle Radar Topography Mission (SRTM) digital elevation model (DEM) which covers and extends beyond the study area of interest was selected, and it was reprojected to UTM Zone 32 North / WGS 84 to better align with the local project requirements. UTM projections are known for their conformal nature, which preserves shapes and angles, making them particularly suited for precise measurements and detailed analyses of specific geographic regions (Kumar et al., 2023).

To address depressions in the DEM and maintain continuous water flow without errors, the fill sink algorithm (Wang & Liu, 2006) from the QGIS 3.4.8 processing modules library was applied. Stream classification relied on the Strahler order algorithm with a threshold value of 8, which allowed the identification of various stream sizes within the study area. Subsequent hydrological parameters, including flow direction, channel networks and drainage basins, were calculated using QGIS 3.4.8 tools, aligning with the Strahler-organized streams. To identify the sub-catchments relevant to the study, an outflow point was assigned to the primary river in the area of interest. The coordinates of this outflow point were determined using the coordinate capture tool within the QGIS 3.4.8 plugin manager. The contributing area to this outflow point was then calculated using the upslope algorithm, also available in QGIS 3.4.8. For a comprehensive understanding of input data and processing, readers are referred to the Supplementary Data section, which includes corresponding figures (Fig. S1 through S7).

### Sample collection and preparation

During the field sampling campaign on August 28th and 29th, 2019, a total of 53 samples were collected from the Kporghor River estuary sub-catchment. These samples included 14 surface sediment samples (0.00 to 0.15 m), 14 subsurface sediment samples (2.0 m), 15 surface water samples and 10 groundwater samples. The sampling stations were selected along a sampling transect that follows the natural hydrological system, with the river channel (Fig. 1) serving as a representative pathway for assessing contaminants, sediments and water-induced transportable materials within the study area. The sampling was performed mainly during outgoing tide and started before 10:00 local time on both days. An Edelman hand auger, with a diameter of 70 mm, was used to collect sediment samples. The collected sediments were immediately placed in pre-cleaned amber glass bottles (60 mL).

The groundwater in the sampled area is relatively shallow, with the static water level encountered at an average depth of 0.7 m at the bottom of each hand-augered boring. To collect groundwater samples, a stainless-steel screen was submerged with 1 m extendable below the static water level. The retrieved groundwater samples were immediately transferred into pre-cleaned laboratory supplied amber glass bottles (250 mL). To prevent contamination from previous sampling points, the sampling screen and its extendable arm were decontaminated with freshwater and detergent between each sampling location. The glass sampling bottles were filled to minimize the amount of headspace.

To obtain surface water samples, pre-cleaned laboratory supplied amber glass bottles (250 mL) were immersed 0.2–0.3 m below the surface of the river channel. A fabricated aluminium arm, extended to 2 m, was affixed to the opening of the sampling bottle to collect samples from the middle or near the middle of the river channel. Surface water samples were typically collected against the flow direction of the river channel to ensure that they were representative of the water body. The sample bottles were filled to minimize the headspace. To prevent contamination between sampling points, the 2-m extendable arm was washed between samples with freshwater and detergent.

Following completion of each sampling cycle, all obtained samples were immediately labelled and placed into corresponding cooling boxes equipped with ice packs for transport into and from the field. All samples arrived at the analytical laboratory in ice boxes (maintaining temperatures at or below 4 °C.) within 10 h of field sampling.

### Chemicals

Analytical reagent grade dichloromethane, methyl-chloride, n-hexane, deionized water and anhydrous sodium sulphate from Merck (Darmstadt, Germany) were utilized in the studies. The internal standards consisted of seven deuterated PAHs including naphthalene-d8, biphenyl-d10, phenanthrene-d10, pyrene-d10, benzo[a]athracene-d10, benzo[a]pyrene-d12 and benzo[g,h,i]perylene-d12. O-terphenyl was mainly used as the surrogate standard. The internal and surrogate standards were purchased from Neochema GmbH (Bodenheim, Germany).

### Sediment and water analysis

The sediment extraction procedures were performed according to methods described by Haleyur et al. (2016) with small modifications for our samples. Sediment samples (5 g) were weighed into 50-mL glass centrifuge tubes, and then dried in an oven at 50 °C for 24 h to remove any moisture. Internal standard solution was added to each tube. The internal standard solution consisted of 50 ng/mL each of naphthalene-d8, biphenyl-d10, phenanthrene-d10, pyrene-d10, benzo[a]athracene-d10, benzo[a]pyrene-d12 and benzo[g,h,i]perylene-d12 in dichloromethane. The samples were spiked with surrogate standard (O-terphenyl) at a concentration of 100 ng/g. Then, 10 mL of dichloromethane was added to each tube, and the samples were extracted using accelerated solvent extraction (ASE) with the following conditions: temperature, 100 °C; pressure, 1500 psi; static time, 5 min; flush volume, 60% and purge time, 120 s.

For water samples, the extraction procedure followed methods by Adeniji and et al., (2017a, b). Internal standard solutions described for the sediments were added to the sample at a final concentration of 50 ng/mL. The surrogate standard (O-terphenyl) for water samples was added at a concentration of 1 μg/L. The sample was extracted using liquid–liquid extraction with 30 mL of dichloromethane. The mixture was shaken for 2 min and then centrifuged at 2500 rpm for 5 min. The organic solvent layer was transferred to a clean separatory funnel and the extraction was repeated two more times. The combined organic extracts were then evaporated to dryness using a rotary evaporator under nitrogen at 40 °C. The residue was reconstituted in 1 mL of n-hexane and the extract was cleaned up by passing through a silica gel column.

### Chemicals analysis

All extract samples were analyzed for total petroleum hydrocarbons (TPH) and polycyclic aromatic hydrocarbons (PAHs) using an Agilent 6890N Network gas chromatograph (GC) equipped with a flame ionization detector (FID). The TPH content, covering a range of C10–C40, was determined in water and sediment samples following the US EPA 8015C standard method. The analysis of target PAHs, specifically the 16 U.S. EPA-regulated PAHs and seven deuterated PAHs, in water and sediment samples, followed the US EPA 8270D standard method. Consequently, GC and FID operating conditions were modified for TPH and PAH analyses based on the standard method being applied to the extracts (i.e. 8015C or 8270D). The following section describes the general operating conditions. Helium was used as the carrier gas, and the analysis was performed using an HP-5 column (30 m × 0.32 mm × 0.25 μm). The detector temperature was set at 350 °C, with a hydrogen gas flow rate at 35 mL/min, air flow rate at 350 mL/min and helium gas flow rate at 20 mL/min. The inlet operated with an electronic pneumatic capture splitless make, with a temperature of 275 °C, pressure of 14.8 psi, split flow rate of 6.8 mL/min and total flow rate of 25.8 mL/min. The oven temperature ranged from 65 to 325 °C, with a run time of approximately 53.5 min, pressure set at 14.8 psi and a flow rate of 3.3 mL/min.

### Quality control

Quality control was carried out by spiking surrogate standard into each water and sediment sample before extraction and accepting recoveries within the range of 71–102%. For standard procedural purposes and to maintain sediment and water samples integrity, data on all collected samples were inventoried into a field datasheet (completed during field sampling) and a standard chain of custody (filled-in upon sampling completion).

## Results and discussion

### Sub-catchment delineation

Figure 1 represents the final output of the sub-catchment delineation process. It shows primarily the surface flow architecture of the Kporghor River Estuary sub-catchment, characterized by a main river channel (Strahler order 1) fed by two tributaries (Strahler order 2).

This approach offers a hydrological system defined sampling transect where the spatio-temporal variability of petroleum contamination is better captured for the assessment of current contamination levels and the monitoring of subsequent petroleum removal processes, ecological status and other restoration indicators (Erunova & Yakubailik, 2023; Wei et al., 2023).

The sub-catchment approach allows the classification of petroleum pollution sources into internal and external categories, aiding tailored pollution control strategies. For instance, during fieldwork in the Kporghor River Estuary sub-catchment, two sources were identified: internal (disused oil cooking pits) and external (river channel-transported crude oil). This type of pollution source classification is critical for guiding environmental protection decisions and developing pollution control mechanisms when responding to petroleum contamination within the Delta.

While the sub-catchment method introduced in the current study represents a novel approach to assessing and managing petroleum contamination in the Niger Delta, it is essential to contextualize this method within the broader domain of catchment-based assessments used in environmental science. While direct comparisons to studies employing a similar approach for oil contamination are limited, the catchment-based approach has found applications in monitoring and managing various environmental pollutants across different regions. For instance, research has shown that catchment-based assessments have been successfully used to evaluate the effects of nutrient and pesticide pollutants on aquatic ecosystems (Bowmer, 2013; Teklu et al., 2018). Similarly, studies focusing on plastic pollution have adopted a catchment-scale perspective to analyze the transport and distribution of plastic waste within river catchments (Windsor et al., 2019). Additionally, a UK-based case study has emphasized the importance of catchment-based policy approaches in mitigating agricultural water pollution (McGonigle et al., 2012).

Moreover, studies conducted in regions with similarities to the Niger Delta, such as high-energy environments like estuaries, coastal areas and tributaries, have also employed catchment-based assessments to understand pollutant transport and ecological impacts. For example, an approach to catchment-scale groundwater nitrate risk assessment in Northern Ireland (Wang & Yang, 2008) and an ecological assessment of river networks (Kuemmerlen et al., 2019) have utilized catchment-based methods. These studies, while different in pollutant focus and geographical location, share the commonality of high-energy environments where pollutant transport dynamics can be complex and interconnected.

It is important to acknowledge that the sub-catchment method, as applied in the current study, may have unique strengths and weaknesses compared to traditional methods commonly used in the Niger Delta. Traditional grid-based assessments typically rely on arbitrary divisions of the environment, often centered around the immediate spill source area. These assessments serve as rapid screening tools, enabling detailed analysis of contaminant concentrations within narrowly defined zones. While they excel in providing precise information about pollution hotspots, these assessments may have limitations in capturing the broader spatial and temporal variability of petroleum contamination within the complex hydrological landscape of the Niger Delta. In contrast, the sub-catchment-based approach takes a more holistic view, establishing system boundaries based on the natural flow of hydrological systems and ecological criteria. This method considers the interconnected nature of the Delta’s hydrology, recognizing that oil contamination does not adhere to arbitrary boundaries. It provides a framework that encompasses entire sub-catchment areas, reflecting the intricate network of rivers, streams and wetlands within this high-energy environment. Having defined the sub-catchment boundaries and elucidated the rationale behind the approach, the assessment of petroleum contamination within this delineated sub-catchment is the next topic of discussion. The description below outlines the sampling conducted at various stations within the sub-catchment to evaluate the extent of petroleum contamination.

### Sediment samples

#### TPH levels in surface sediment

The concentrations of TPH in the top layer of the sediments varied between 129 to 20,600 mg/kg, with an average value of 7290 mg/kg (SI Table S1). Further information regarding TPH values and relevant summary statistics can be found in SI Table S1 and the accompanying text. There was not a noticeably strong trend in the surficial sediment TPH spatial distributions; however, in general, there are marked differences in TPH concentrations within short distances. Elevated TPH levels were more frequently observed downstream in areas directly influenced by tidal flow, with levels generally exceeding 1000 mg/kg except at specific sampling points including TB3, TB7 and ND1. Based on the extensive surface crude accumulation observed at the shoreline in the downstream area, it is surprising to find relatively low TPH values at the sampling locations TB3, TB7 and ND1. However, this could be partially explained by the variation in spatial energy levels, such as wind, waves and tides, across the investigated area. It is likely that these three sampling locations experience low energy levels, which could limit the emulsification of petroleum resulting in reduced spreading and mixing into the sediment (Bonte et al., 2020; Nwipie et al., 2019).

The TPH concentrations encountered in the surface sediment samples in this study are consistent with those reported in multiple locations within the Niger Delta (Lindén & Pålsson, 2013). However, the TPH concentrations in the surface sediment samples collected from the contaminated mangrove areas in Bodo, Niger Delta, were higher than those observed in this current study (Bonte et al., 2020; Gundlach et al., 2021; Little et al., 2018; Saunders et al., 2022). In comparison to other petroleum-impacted marine environments fed by riverine sources, the TPH concentrations reported in this study are similar to those found in the urban tributaries of the River Clyde in Glasgow, UK (Vane et al., 2017).

In 1991, the Federal Agency Department of Petroleum Resources (DPR) in Nigeria issued the Environmental Guidelines and Standards for the Petroleum Industry in Nigeria (EGASPIN), which has undergone revisions and updates in 2002, 2016 and 2018 (Olawuyi & Tubodenyefa, 2018). EGASPIN represents the major operational guidance for environmental regulation and management of Nigeria’s oil industry. It provides environmental monitoring criteria mainly through defined target and intervention values for indicator petroleum parameters including TPH, PAHs and BTEX (i.e. benzene, toluene, ethylbenzene and xylene). EGASPIN’s regulatory intervention values for TPH and PAHs in sediment are 5000 mg/kg and 40 mg/kg, respectively (DPR, 2002).

**Fig. 2:**
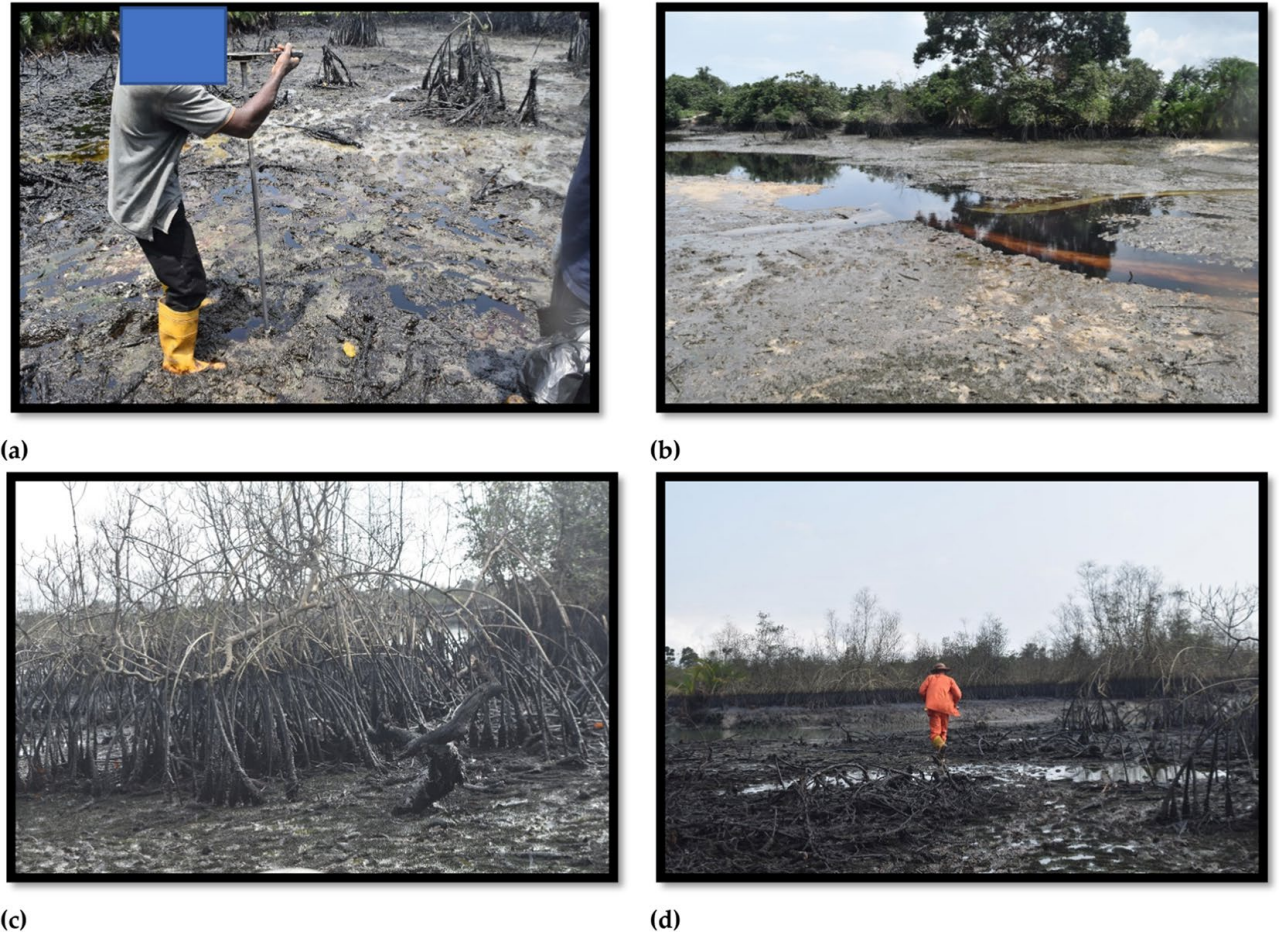
Petroleum contamination in the study area: **a** heavily oiled creek sediment exposed at the low tide mark, **b** floating crude oil at the low tide mark, **c** impacted mangrove shoots, and **d** mangrove substrates covered in oil with heavily impacted areas in the background

Based on the comparison of TPH concentrations in the surficial sediment reported in this study with EGASPIN’s regulatory intervention value and other proposed sediment pollution guidelines (Massoud et al., 1996; Pinedo et al., 2013), it can be inferred that the investigated area is heavily polluted over large areas. This chronic contamination is likely to have current and potential impacts on the sub-catchment area. Specifically, the sub-catchment’s main river channel-bed, shoreline sediments and fringe mangrove vegetation (Fig. 2) are expected to be the most vulnerable ecological communities to physical oil smothering and toxicological effects.

The impact of smothering on the community of organisms living in the sediment within the study area is unclear. However, based on observations of heavily oiled sediment in the main river channel and along the shoreline when exposed at low tide (Fig. 2), as well as the presence of black tar indicating a long-standing pollution, it is reasonable to assume that smothering may have had a negative impact on the organisms living in the sediment (Daly et al., 2016; Lin & Tjeerdema, 2008).

The loss of large areas of mangrove (Fig. 2) and the current heavy oil coating on the roots and remains of the dead mangroves are indicative of a combined smothering and toxic effect on the investigated area’s vegetations. As compared with lighter and medium (diesel) oil, heavy oils such as crude generally cause less toxic effects on plant vegetation including mangroves (Michel & Fingas, 2016). However, when oil accumulates in the sediment and remains for extended periods, mangrove roots experience a continuous exposure which often results in complete loss of mangrove areas (Dick & Osunkoya, 2000; NRC, 2003).

#### TPH levels in subsurface sediment

The deeper layer sediment samples exhibited a wide range of TPH concentrations, ranging from 15.5 to 985 mg/kg, with a mean value of 195 ± 12.34 mg/kg (SI Table S1). The majority of TPH levels in the deeper layer sediment were below 180 mg/kg, with notable exceptions observed at sampling points TB7, ND5 and ND8, where comparatively elevated TPH concentrations reaching several hundred mg/kg, were recorded (SI Table S1). In general, except for TB7, the TPH concentrations in the subsurface sediment samples were substantially lower than those in the corresponding surface sediment samples (SI Table S1).

Based on the quantitative analysis of TPH concentrations in subsurface sediment samples, in which TPH levels exceeding several hundred mg/kg were observed in the more contaminated samples, it is evident that petroleum contamination has extended to at least 2-m depth within the investigated area. The TPH concentrations measured in the deeper sediment layer in this study are generally lower when compared to the findings from a limited number of existing studies (Akpokodje, 2018; UNEP, 2011) that have assessed petroleum contamination in deeper sediment layers of the Niger Delta region.

#### PAH levels in surface sediment

**Fig. 3:**
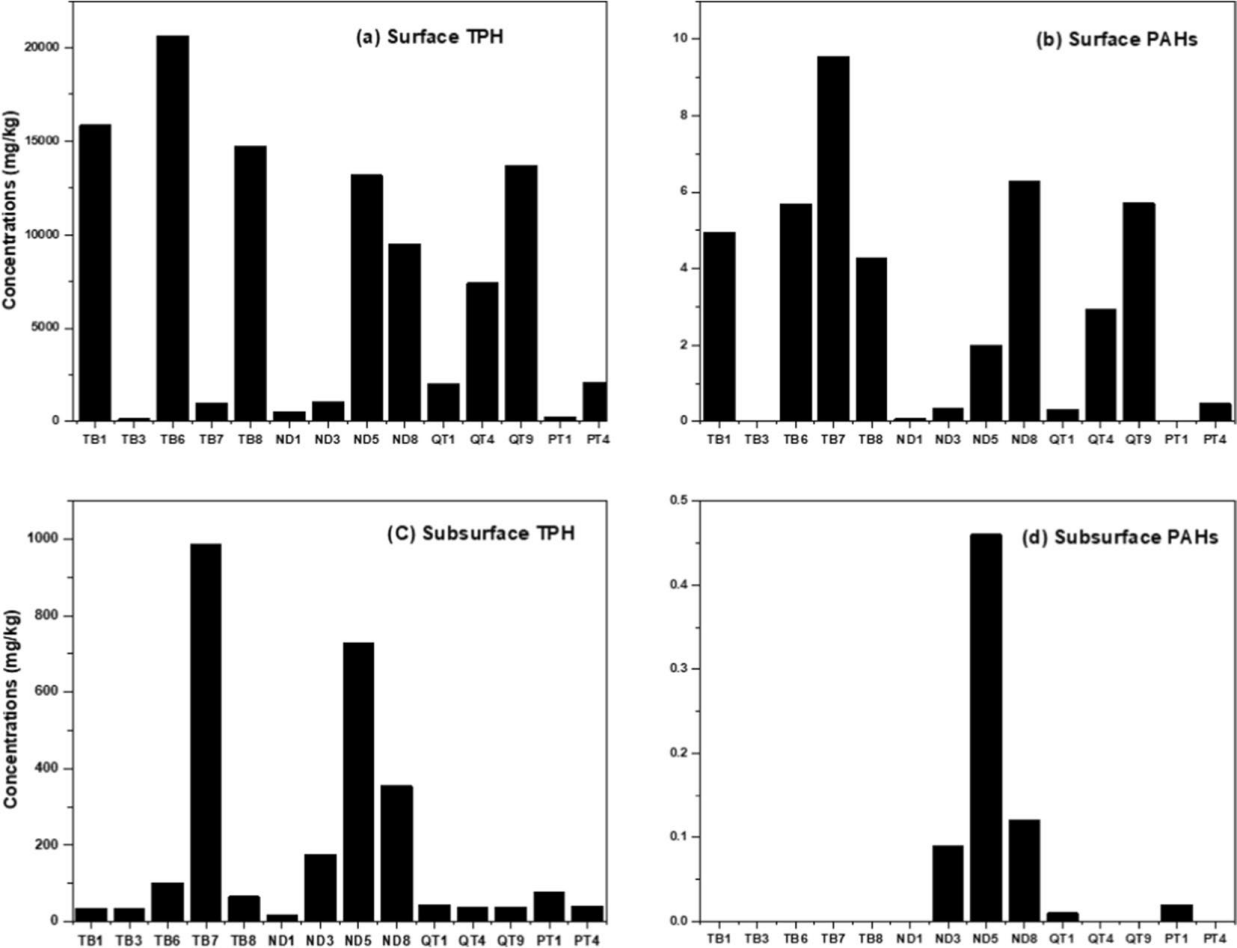
Distribution of TPH and ∑PAHs in Kporghor River Estuary sediment: **a** surface TPH, **b** surface PAHs, **c** subsurface TPH, and **d** subsurface PAHs

The concentrations of ∑16 PAHs in surficial sediments were quantified, ranging from 0.07 to 9.55 mg/kg with a mean concentration of 3.55 ± 0.81 mg/kg (SI Table S1). Notably, the highest concentration of PAHs was observed at sampling point TB7 (Fig. 3).

**Fig. 4:**
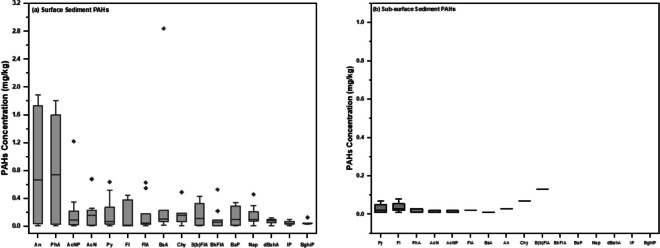
Comparative analysis using a whisker plot of sediment samples for individual PAH concentrations sorted by abundance: **a** surface sediment PAHs and **b** subsurface sediment PAHs. Error bars reflect the range of non-outlying values, the horizontal line is the median, and black diamonds are outliers

Anthracene and phenanthrene were each detected at a frequency of 93%, indicating high occurrence, while benzo (g, h, i) perylene exhibited the lowest detection frequency at 43% (SI Table S2, Fig. 4).

The distribution pattern of PAHs in the surficial sediments revealed that low molecular weight (LMW) PAHs with 2–3 rings accounted for 65% of the total concentration of 16 PAHs, whereas high molecular weight (HMW) PAHs with 4, 5 and 6 rings constituted 35% of the total. This distribution of PAHs is consistent with a petrogenic source of PAHs (Burgess et al., 2003a, b).

As previously stated, the regulatory intervention value for PAHs in sediment, determined by EGASPIN, stands at 40 mg/kg. However, it is worth noting that EGASPIN only covers 10 out of the 16 PAHs (Bonte et al., 2020) and primarily focuses on oil and chemical pollution control rather than biodiversity and ecological management (Little et al., 2018). Against this background, the potential risks associated with PAHs in the sediments of the Kporghor River Estuary were evaluated using the risk coefficient (RQ). RQ_(NCs)_ and RQ_(MPCs)_ were calculated using Eqs. (1) and (2) (Cao et al., 2010; Kalf et al., 1997):

1 $${RQ}_{NCs}=\frac{{C}_{PAHs}}{{C}_{QV\left(NCs\right)}}$$

2 $${RQ}_{MPCs}=\frac{{C}_{PAHs}}{{C}_{QV\left(MPCs\right)}}$$

where C_PAHs_ represents the measured concentration of PAHs, and C_QV_ denotes the concentration associated with a particular effect. NCs denote negligible concentrations, while MPCs indicate maximum permissible concentrations. The NCs and MPCs values for individual PAHs in the sediments, along with their ecological risk classifications, are detailed in Table 1.

**Table 1:** Ecological risk classification of individual PAHs and ∑PAHs based on approaches described by Cao et al. (2010) and Kalf et al. (1997)

Individual PAH			∑PAHs		
RQ_(NCs)_	RQ_(MPCs)_	Risk level	RQ ∑PAHs_(NCs)_	RQ_∑PAHs(MPCs)_	Risk level
0	–	Risk free	0	–	Risk free
			≥ 1; < 800	0	Low
≥ 1	< 1	Moderate	≥ 800	0	Moderate
			< 800	≥ 1	Moderate
–	≥ 1	High	≥ 800	≥ 1	High

A RQ_(NCs)_ below 1.0 indicates negligible concern, while an RQ_(MPCs)_ exceeding 1.0 suggests severe risk of contamination by single PAHs. If RQ_(NCs)_ surpasses 1.0 and RQ_(MPCs)_ remains below 1.0, it signifies a moderate level of risk due to the proportion of contaminated PAHs. The mean RQ_(NCs)_ values for individual PAHs exceeded 1.0 at all sampling stations except TB 7 and PT 4 (SI Table S3). The mean RQ_(MPCs)_ ranged between 0.02 to 2.72 for all stations except TB 7 and PT 4 (SI Table S4). For individual PAHs, moderate to high risks are anticipated in the former stations, while the latter stations are deemed risk free. The RQ_∑PAHs(NCs)_ and the RQ_∑PAHs(MPCs)_ of Kphorghor River Estuary sediment are illustrated in Fig. S8. In the figure, the observation that RQ_∑PAHs(NCs)_ are below 800 and RQ_∑PAHs(MPCs)_ values are below 1 in all stations indicate a low to moderate level of risk.

Beyond the RQ assessment, a comparison of PAH concentrations in sediment to established sediment quality guidelines (SQGs), specifically the Effect Range Low (ERL) and Effect Range Median (ERM), was conducted. These guidelines define concentrations correlated with the occurrence frequency of adverse biological effects on sediment-dwelling organisms (Long et al., 1995). Interestingly, findings revealed that 57% of the surficial sediment samples had ∑16 PAH levels below the ERL of 4 mg/kg, while 43% exceeded the ERL, with none reaching the ERM of 44 mg/kg. These results suggest that the surficial sediment in the investigated area exhibits mild PAH contamination, with occasional probable adverse effects on sediment-dwelling organisms.

The findings of this study regarding the levels of PAHs in surficial sediment are consistent with previous studies conducted in multiple locations across the Niger Delta (Lindén & Pålsson, 2013). When compared globally, the PAH levels reported in this study are similar to surficial sediments observed in the Gironde Estuary, France (Budzinski et al., 1997), Suez Gulf (Nemr et al., 2005), the Danube River (Škrbić et al., 2005) and rivers in Tianjin, China (Shi et al., 2005). However, the surficial sediment concentrations in this study were lower than those found in the Glasgow urban tributary (Vane et al., 2017), Boston Harbor (USA) (Wang et al., 2001), the Yangtze River Estuary (Jiang et al., 2011), Nansi Lake, China (Li et al., 2009), Samsun Coast, Turkey (Tepe & Taştekin, 2022) and Giresun Coast, Turkey (Tepe et al., 2022).

#### PAH levels in subsurface sediment

The concentrations of ∑16 PAHs in the subsurface sediment samples were mostly below detection limits, except at four sampling points (ND3, ND5, ND8 & PT1) where values ranged from 0.02 to 0.46 mg/kg. Applying the risk quotient (RQ) to the subsurface sediment samples from the Kphorgor River Estuary indicated a lack of risk. Furthermore, the concentrations of individual PAHs in the subsurface sediment were all below the EGASPIN and ERL values, indicating no adverse biological effects in the deeper sediment layer of the investigated area.

### Water samples

#### TPH levels in surface water

The measured TPH concentrations in the surface water of the main river channel were consistently high, with values ranging from 103 to 620 mg/L and a mean of 205 ± 32.28 mg/L (SI Table S5). The spatial distribution of TPH in surface waters revealed a discernible trend, with substantially higher levels downstream, elevated levels in the midstream, and another peak of very high levels in the upper reaches of the sampled area.

**Fig. 5:**
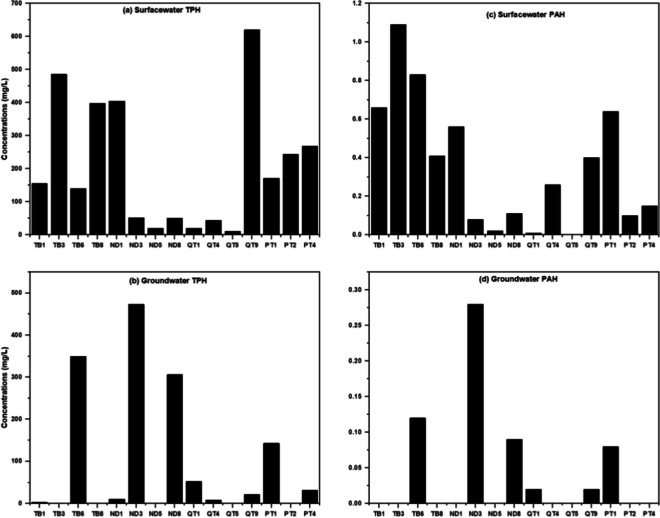
Distribution of TPH and ∑PAHs in Kporghor River Estuary surface and groundwaters: **a** surface water TPH, **b** groundwater TPH, **c** surface water PAHs, and **d** groundwater PAHs

The consistently high concentrations of TPH in the surface water, as observed in this study (Fig. 5), are not surprising considering the visible presence of floating petroleum at the low and high tide mark (Fig. S9). These sheens consist of a mixture of fresh oil input and resuspended stranded shoreline oil, as depicted in Figure S9. The elevated TPH levels also suggest the existence of recent and ongoing sources of petroleum pollution. The concentrations at the lower reach of the sub-catchment suggest this area experiences direct impact from main pollution source inputs (e.g. tidal mobilized crude) while the high levels at the middle reaches of the sub-catchment probably indicates increased dilution effects in this area.

Currently, EGAPSIN has no regulatory values for petroleum in surface water; however, the surface water TPH mean value reported here is nearly 685 times higher than the European Union (EU) petroleum threshold value of 300 μg/L for estuary and harbour basin water (Adeniji et al., 2017a, 2017b; Sciortino and Ravikumar, 1999). These exceptionally high values are indicative of chronic and deleterious exposure even to the most tolerant water dwelling organisms and are particularly dangerous for human exposure and consumption. The surface water TPH levels observed in this study are close to those encountered across other sites in the Niger Delta (Lindén & Pålsson, 2013). Additionally, coastal water samples from Terrebonne Bay, Louisiana (USA), have shown TPH levels approaching those observed in this study (Sammarco et al., 2013).

#### TPH levels in groundwater

The analysis of groundwater samples revealed elevated TPH concentrations, with an average value of 134 ± 37.53 mg/L and a range of 31.8 to 473 mg/L as presented in SI Table S5. The distribution of TPH in groundwater is characterized by a patchy pattern, with no discernible spatial trend (Fig. 5). Additionally, there is no strong correlation (Pearson’s *r* ≈ 0.346) observed between TPH levels in surficial sediment and the corresponding contamination levels in groundwater. This lack of correlation and the patchy distribution of TPH in groundwater may be attributed to the heterogeneity in oil input across the study area and non-uniform leaching of petroleum into the groundwater.

The TPH levels detected in the groundwater samples were substantially higher than the regulatory intervention value of 600 μg/L set by EGASPIN. For example, the mean value of TPH in the groundwater samples, which was 140 ± 27.59 mg/L, exceeded EGASPIN’s intervention value by 233 times. These elevated levels indicate widespread and chronic contamination of the groundwater in the investigated area. With levels reaching several hundred mg/L, it is highly likely that the stability and integrity of the biological community in the groundwater has been adversely impacted. The TPH concentrations observed in the highly contaminated groundwater samples in this study are substantially higher than those reported in previous surveys conducted in various areas of the Niger Delta (Alinnor et al., 2014; Lindén & Pålsson, 2013; Omo-Irabor et al., 2008). This disparity in TPH levels may be attributed, in part, to the different environments investigated. Previous surveys primarily collected groundwater samples from boreholes and hand-dug wells located in upland areas, which are terrestrial environments, whereas the groundwater samples in this current study were obtained from a chronically polluted marine environment.

#### PAH levels in surface water

**Fig. 6:**
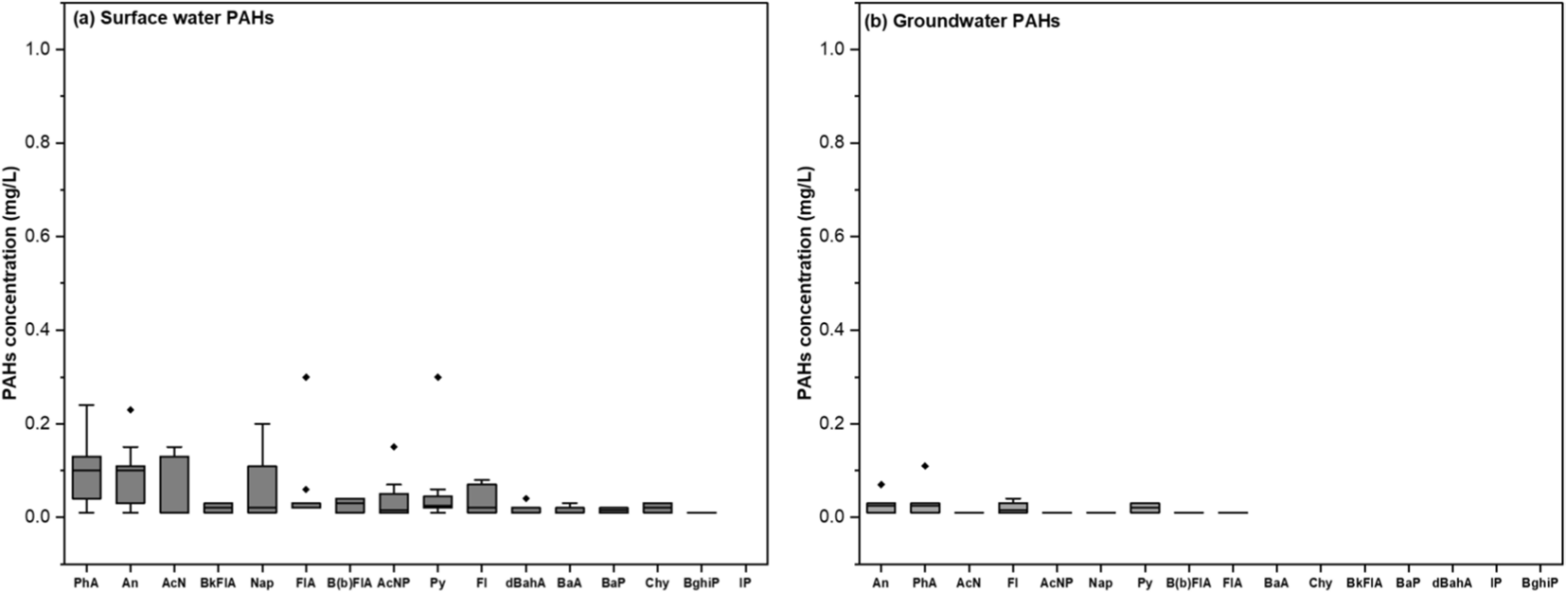
Comparative analysis using a whisker plot of surface water samples for individual PAH concentrations sorted by abundance: **a** surface water PAHs and **b** groundwater PAHs. Error bars reflect the range of non-outlying values, the horizontal line is the median and black diamonds are outliers

The concentrations of ∑16 PAHs (the sum of 16 specific polycyclic aromatic hydrocarbons as defined by the United States Environmental Protection Agency) in surface water samples ranged from below the detection limit (0.01 mg/L) to 1.09 mg/L, with a mean of 0.35 ± 0.21 mg/L (SI Table S5). Analysis of individual PAHs revealed that anthracene (93%) and phenanthrene (87%) were the most frequently detected compounds, while benzo(g, h, i) perylene (7%) and Indeno(1,2,3-cd) pyrene (0%) were the least frequently detected across the sampling locations (SI Table S6, Fig. 6).

The dominant fraction of ∑16 PAHs in the surface water of the investigated area was composed of low molecular weight (LMW) PAHs with 2–3 rings, accounting for 77% of the total, while high molecular weight (HMW) PAHs with 4, 5 or 6 rings were less dominant, comprising the remaining 23%. As noted above, these distributions are consistent with petrogenic PAHs.

The mean concentration (0.35 ± 0.21 mg/L) of Σ16 PAHs in surface water samples from the investigated area substantially exceeds the EU regulatory threshold value for PAHs in River Basins. These remarkably high levels indicate the highly polluted status of the surface water environment in the studied sub-catchment. PAH concentrations exceeding 1 mg/L suggest impairment of the aquatic biota community. Moreover, given that surface water systems serve as a primary source of navigation, agriculture, traditional fish farming and recreation for the local communities within the sub-catchment, there are substantial concerns regarding human health risks.

The concentrations of ∑16 (PAHs) in the surface water reported in this current study exhibit a spectrum of variability, with some concentrations comparable to previous studies conducted in rivers, streams and creeks of the Niger Delta by Lindén and Pålsson (2013) and by (Inam et al., 2016), but far below the levels reported by (Anyakora et al., 2005) and Nganje et al. (2012). In comparison to other similar aqueous environments, PAH levels in the surface water during dry periods in the Liaohe River Basin, China, as reported by Lv et al. (2014), and in the sea-surface microlayer of the Venice Lagoon, Italy, as reported by (Manodori et al., 2006) and Algoa Bay, South Africa (Adeniji et al. 2019) and Rivers in Tianjin, China (Shi et al., 2005) and the Luanhe River Basin, China (Li et al. 2010) were substantially lower than the levels encountered in the more PAH-contaminated samples within the current study.

#### PAH levels in groundwater

The groundwater samples exhibited a wide range of ∑16 PAH concentrations spanning from below the detection limit (< 0.01 mg/L) to 0.28 mg/L, with a mean concentration of 0.06 ± 0.03 mg/L (SI Table S5). The ∑16 PAH levels displayed variability among the samples, and their spatial distribution demonstrated inconsistency, resembling that of groundwater TPH. Notably, LMW PAHs (PAHs with 2–3 rings) were the dominant fraction, accounting for 77% of the ∑16 PAHs, while their HMW counterparts (PAHs with 4, 5, 6 rings) constituted only 11% of the total, as shown in SI Table S6 and Fig. 6.

The more PAH-contaminated groundwater samples had levels ranging from 0.08 to 0.28 mg /L, these values appear substantially low when compared with surface water PAH results. This disproportion maybe due to the high sorption tendencies of PAHs to soil/sediment organic matter (Cornelissen et al., 2006) and their increased attenuation processes in the vadose zone (Yang et al., 2013), thus limiting the PAHs reaching the water table. The ∑16 PAH concentrations encountered in three groundwater samples exceeded EGASPIN’s intervention value (81.5 μg/L) indicating a mild and non-widespread PAH contamination of underlying groundwater within the investigated area.

In comparison to previous investigations conducted in the Niger Delta (Anyakora & Coker, 2009), the concentrations of PAHs for the more contaminated groundwater samples within this study are higher. In other areas, a study conducted by Li et al. (2017) reported PAH levels (0.01–0.40 μg/L) in groundwater samples from Yellow River Estuary, North China. Similarly, Brindha and Elango (2014) reported PAH levels ranging from below the detection limits to 12.9 μg/L in groundwater samples from metropolitan Chennai, India. Notably, the concentrations of PAHs in both studies were lower than the mean concentration (0.06 ± 0.03 mg/L) reported in the current study.

#### Exceedance map

**Fig. 7:**
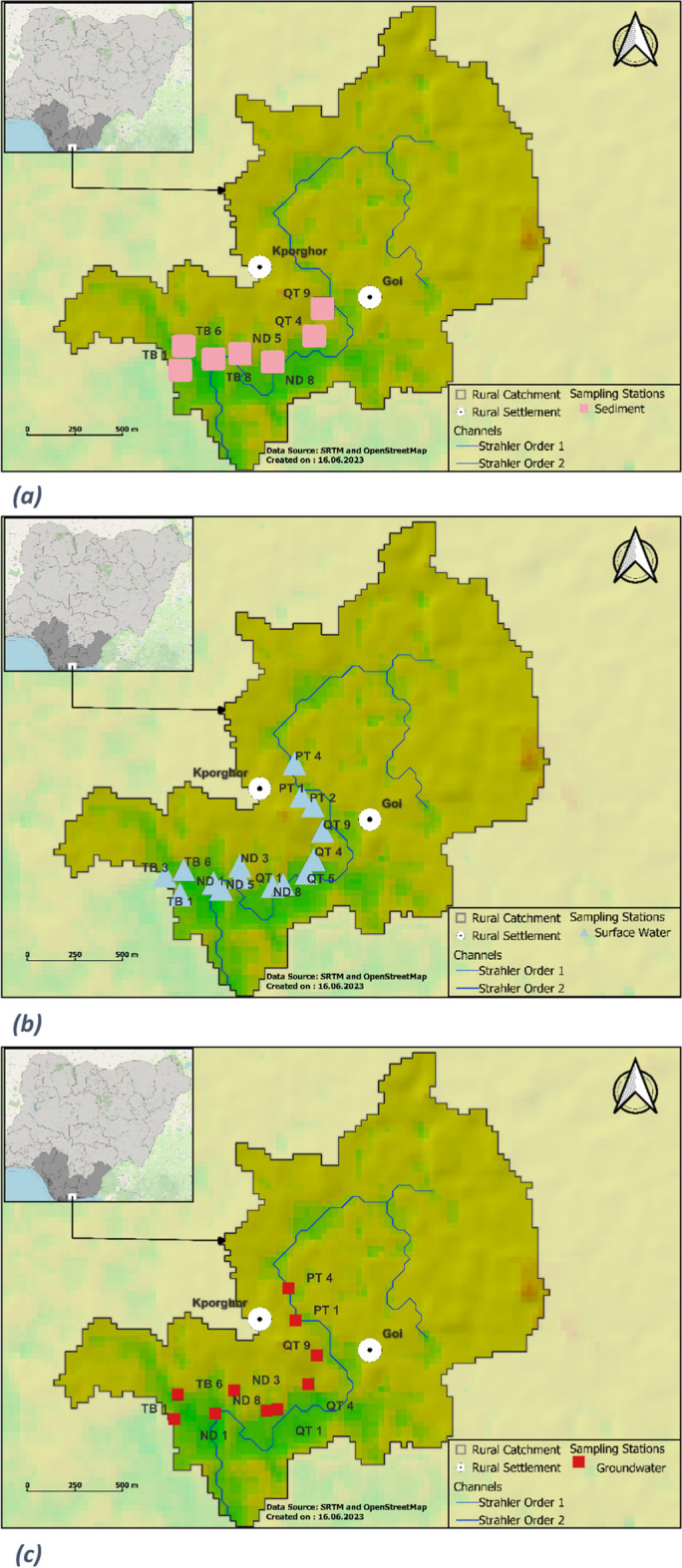
Stations in which **a** surface sediments, **b** surface waters or **c** groundwaters samples exceeded an ecological criterium. Subsurface sediment samples showed no exceedance, therefore are not included in the map

In this section, the exceedance status of sampling stations within the delineated sub-catchment is presented (Fig. 7). These maps highlight the areas where the concentrations of TPH and PAHs exceeded the ecological criteria for specific media including surface sediments, subsurface sediments, surface waters and groundwaters.

As discussed earlier, the sediment criteria for TPH and PAH concentrations were based on the EGASPIN intervention value, RQ, ERL and ERM. Similarly, the European Union (EU) petroleum standards for estuary and harbour basin water were used to evaluate surface water TPH and PAH concentrations. Furthermore, the EGASPIN intervention value was employed to assess TPH and PAH concentrations in groundwater.

The exceedance map provides a visual representation of the areas within the sub-catchment that require particular attention due to elevated concentrations of TPH and PAHs in various media. Stations demonstrating exceedances were evenly distributed around the estuary without any clear geographic pattern. Water stations were the most impacted with 100% of both surface waters and ground waters exceeding a criterium. Sediments were also impacted but to a lesser degree, with 54% of stations exceeding criteria. The distribution of the exceedances reflects a complex combination of the site’s hydrodynamics, the way in which the criteria were derived, and the chemistry of the contaminants. For example, given the hydrophobicity of the TPHs and PAHs more exceedances of the sediment criteria may have been expected (i.e. many of the contaminants will accumulate in the sediments over time). This is one potential topic for future study. Understanding the causes and extent of the exceedances is essential for effectively managing petroleum contamination in the Niger Delta, utilizing a sub-catchment-oriented approach.

## Conclusion

This study proposed the use of a sub-catchment-based delineation approach for assessing petroleum contamination in the Niger Delta region. This approach considers critical parameters specific to the Niger Delta, such as tides, waves, interconnected river systems and groundwater processes, which influence petroleum transport and fate. The assessment of petroleum contamination within the delineated sub-catchment revealed extensive contamination of sediment, surface water and groundwater, originating from spills associated with the refining of crude oil within the investigated sub-catchment, as well as heavy crude oil spills from outside the sub-catchment that were mobilized through river channels. The observations suggest that petroleum contamination has caused extensive damage to mangroves and shoreline vegetation. At the most contaminated sampling points, TPH in sediment reached 20,600 mg/kg, surface water TPH concentrations were up to 620 mg/L, nearly 685 times more than the EU guideline and, in groundwater, TPH concentrations reached 473 mg/L. Greater than 50% of stations exceeded criteria for sediments, and surface and groundwaters. Concentrations and distributions of total and individual PAHs support a petrogenic source. The presence of heavy oil tars observed during field sampling indicates a long history of petroleum contamination, and the heavily impacted mangrove and wetland areas suggests remediation is highly needed and that recovery of the affected area may take decades.

### Supplementary Information

Below is the link to the electronic supplementary material.

Supplementary file1 (DOCX 4794 KB)

## Data Availability

All data supporting the findings of this study in its current form are available within the paper and supplied supplementary materials.
