# Supplementary material for: Assessing petroleum contamination in parts of the Niger Delta based on a sub-catchment delineated field assessment
Source: Environ Monit Assess. 2024 May 29;196(6):585. doi: 10.1007/s10661-024-12743-7 (PMC11136865; doi:10.1007/s10661-024-12743-7)
Supplement: Supplementary file 1 — Supplementary file1 (DOCX 4794 KB) [file 10661_2024_12743_MOESM1_ESM.docx]

**Supplementary Information**

Assessing petroleum contamination in parts of the Niger Delta based on a sub-catchment delineated field assessment.

**Ibukun Olaa***†**, Carsten Drebenstedta, Robert M. Burgess^b^ , Martin Mensah^a^ ,Nils Hotha,Precious Okoroafor^c^, and Christoph Küllsd**

^a^Institute of Mining and Special Civil Engineering, Technical University Mining Academy Freiberg, Gustav-Zeuner Street 1A, Freiberg, 09599, Germany

^b^U.S. Environmental Protection Agency, Office of Research and Development, Center for Environmental Measurement and Modeling, Atlantic Coastal Environmental Sciences Division, 27 Tarzwell Drive, Narragansett, Rhode Island 02882, United States

^c^Institute of Biosciences/Interdisciplinary Environmental Research Centre, Freiberg Technical University of Mining, Leipziger Street 29, Freiberg, 09599, Germany

dLabor für Hydrologie und Internationale Wasserwirtschaft, Technische Hochschule

Lübeck, Lübeck, 23562, Schleswig-Holstein, Germany


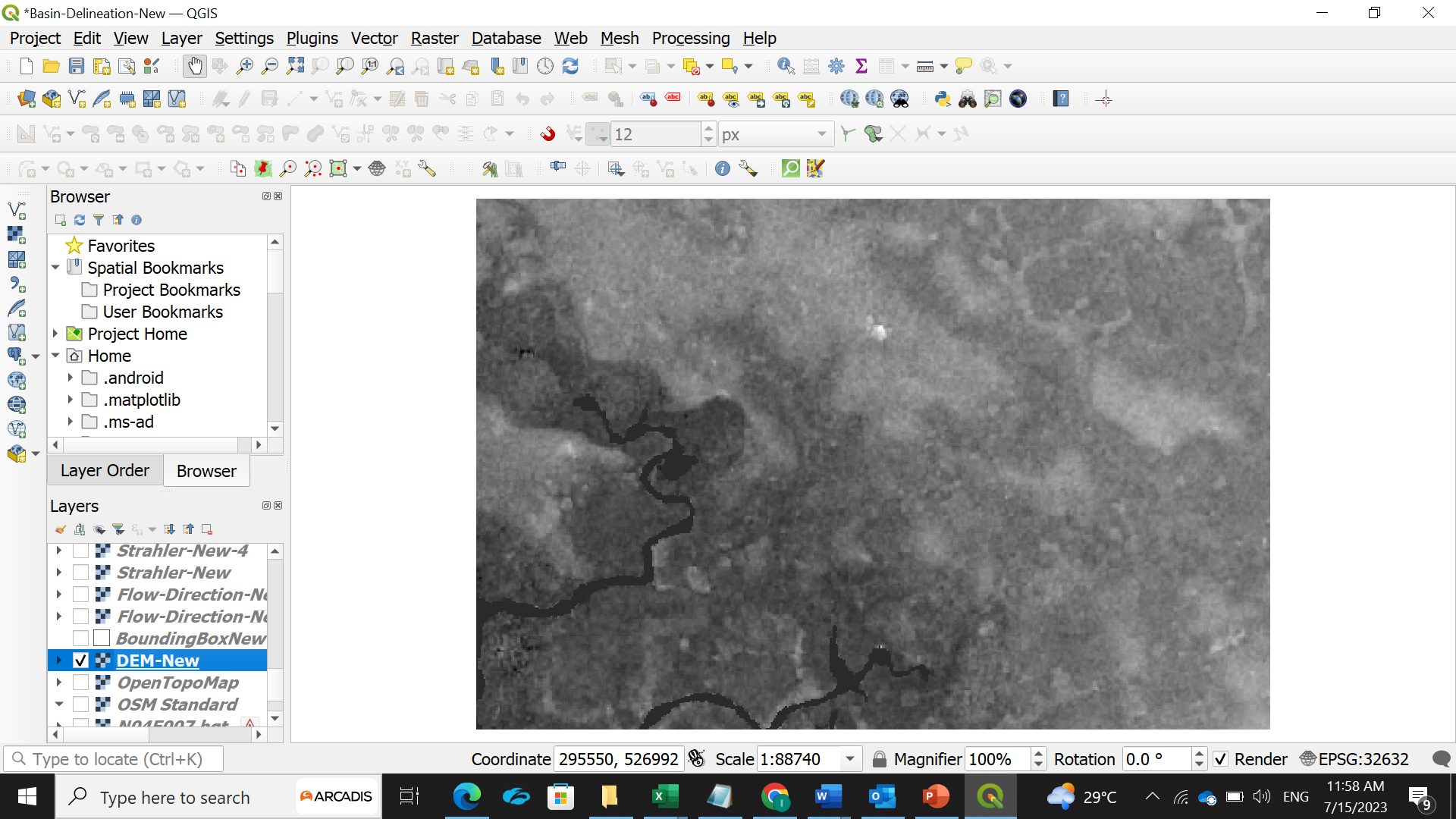


**Fig. S1** Digital elevation model (DEM) downloaded from <https://earthexplorer.usgs.gov/>


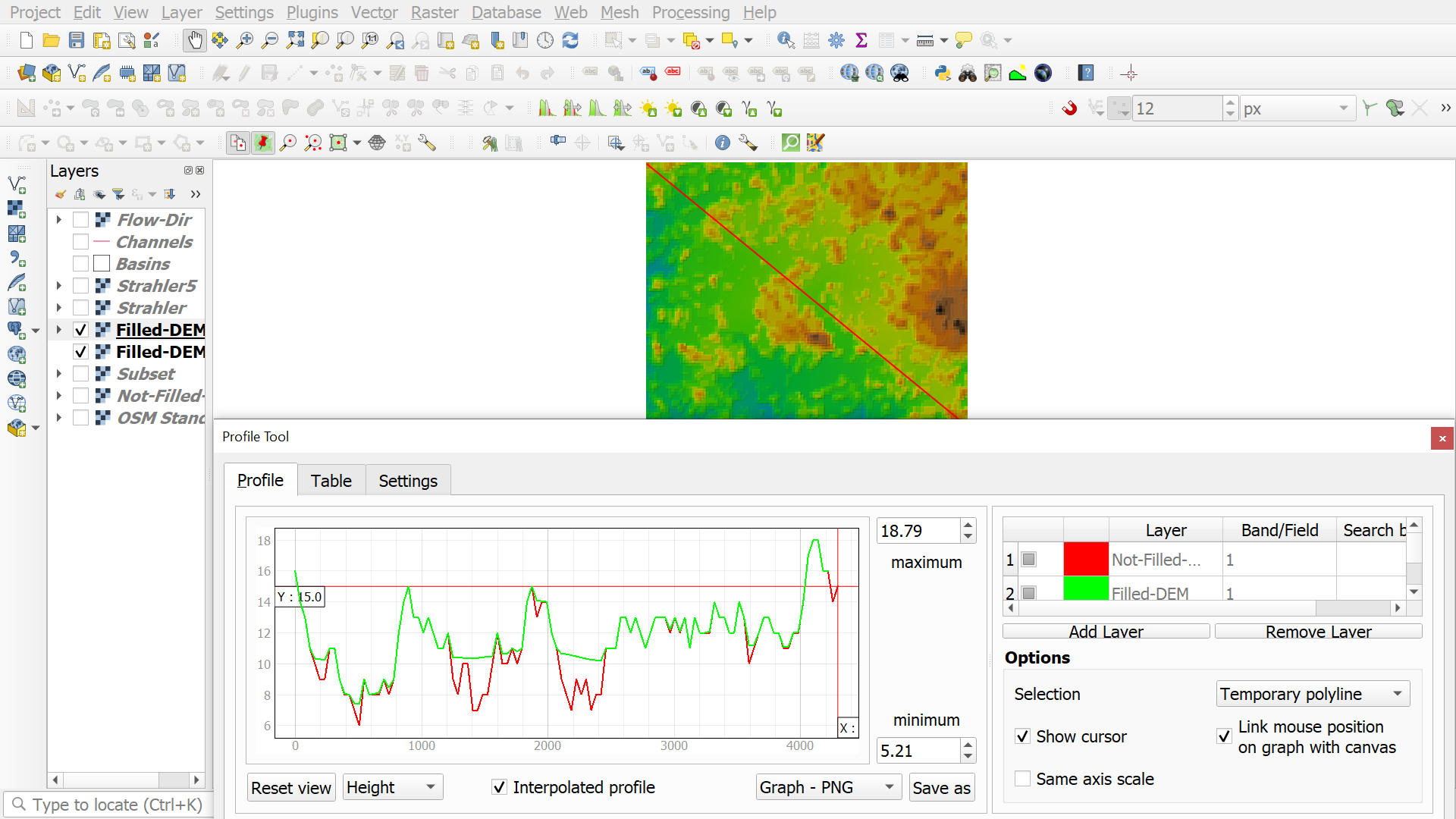


**Fig. S2** Filled DEM using the fill sink algorithm developed by Wang and Liu (2006)


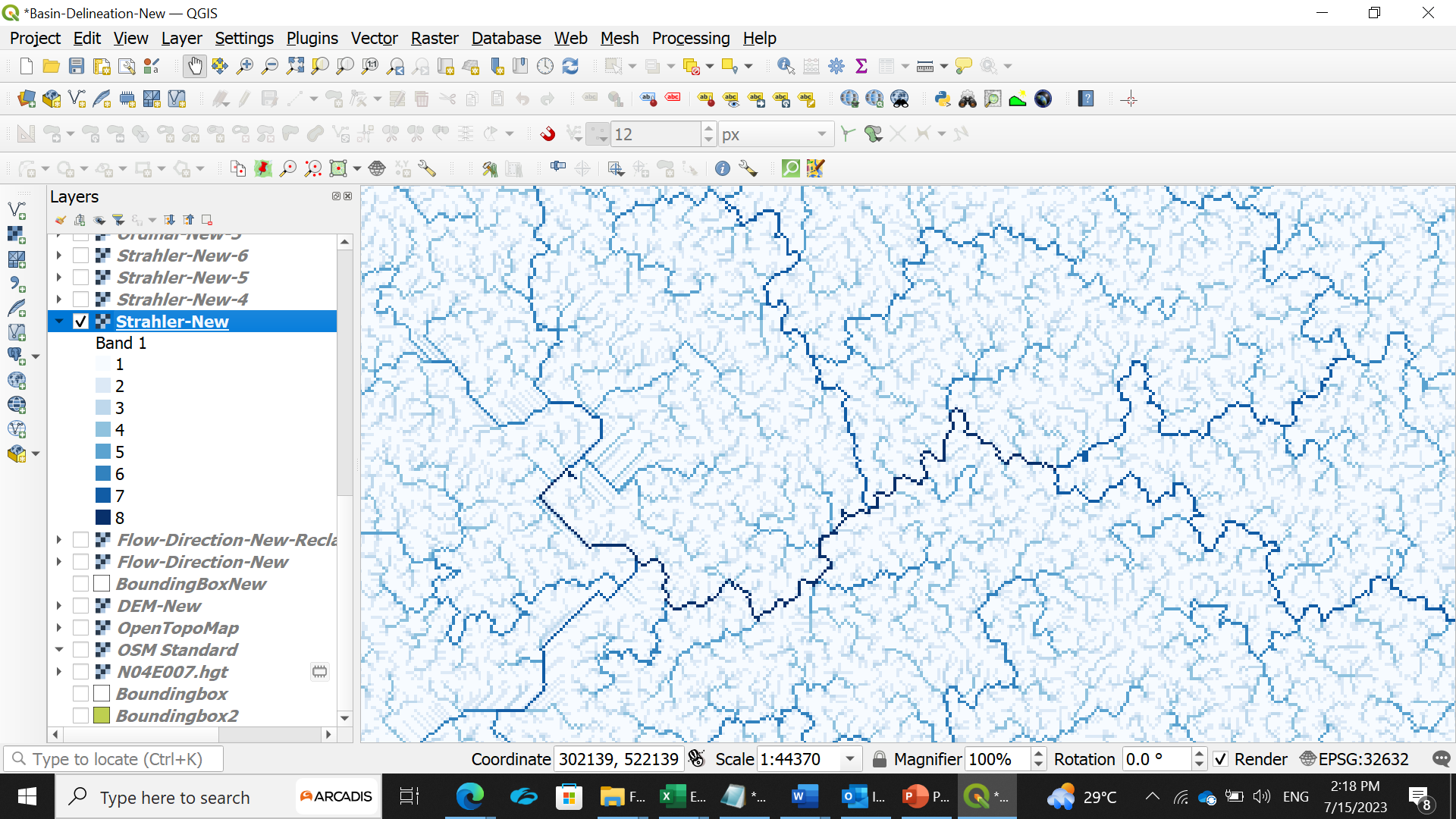


**Fig.S3** Strahler orders calibration using the Strahler order algorithm available in QGIS 3.4.8 processing modules library


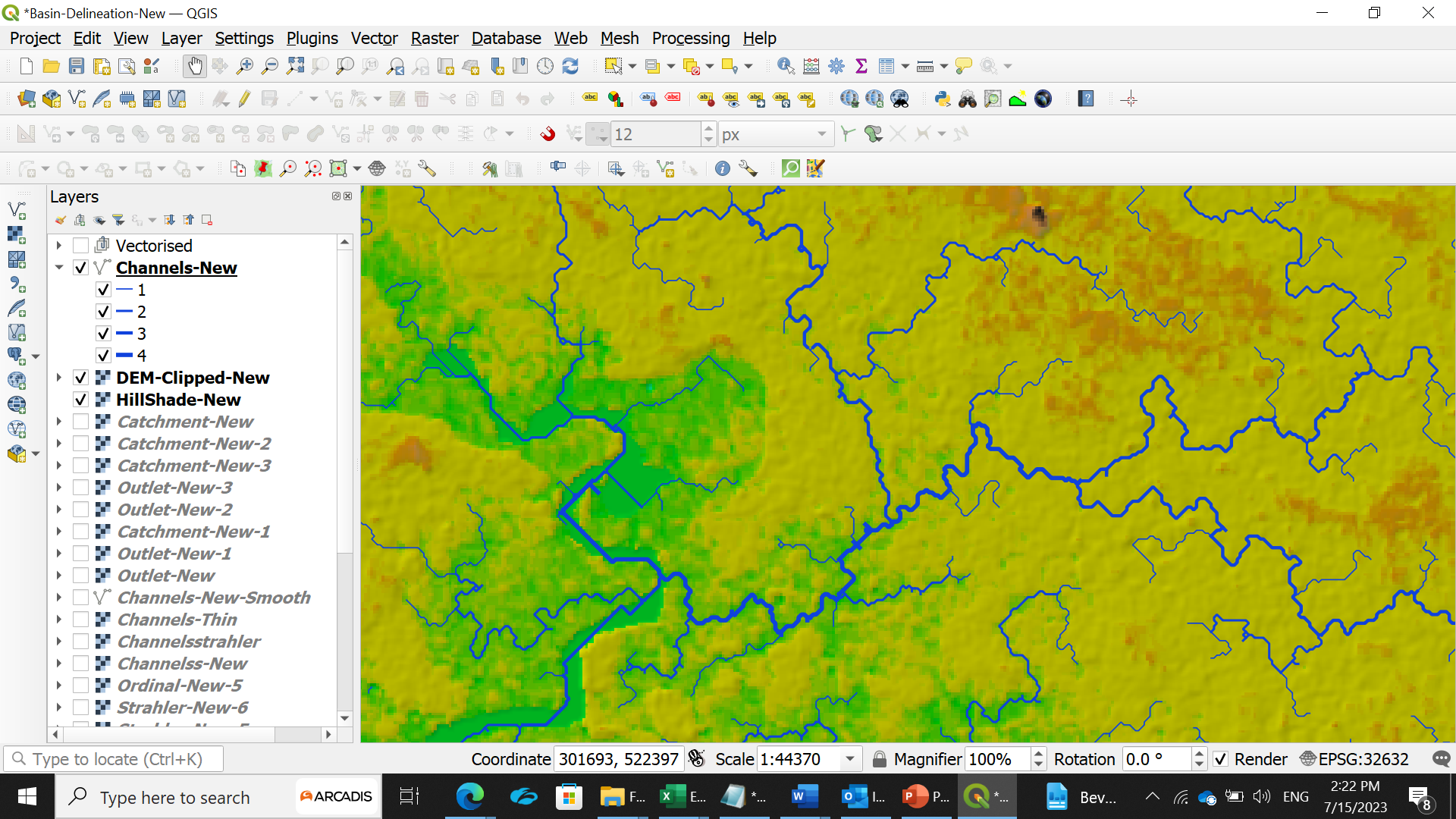
**Fig. S4** River network derivation using channel network algorithm available in in QGIS 3.4.8 processing modules library


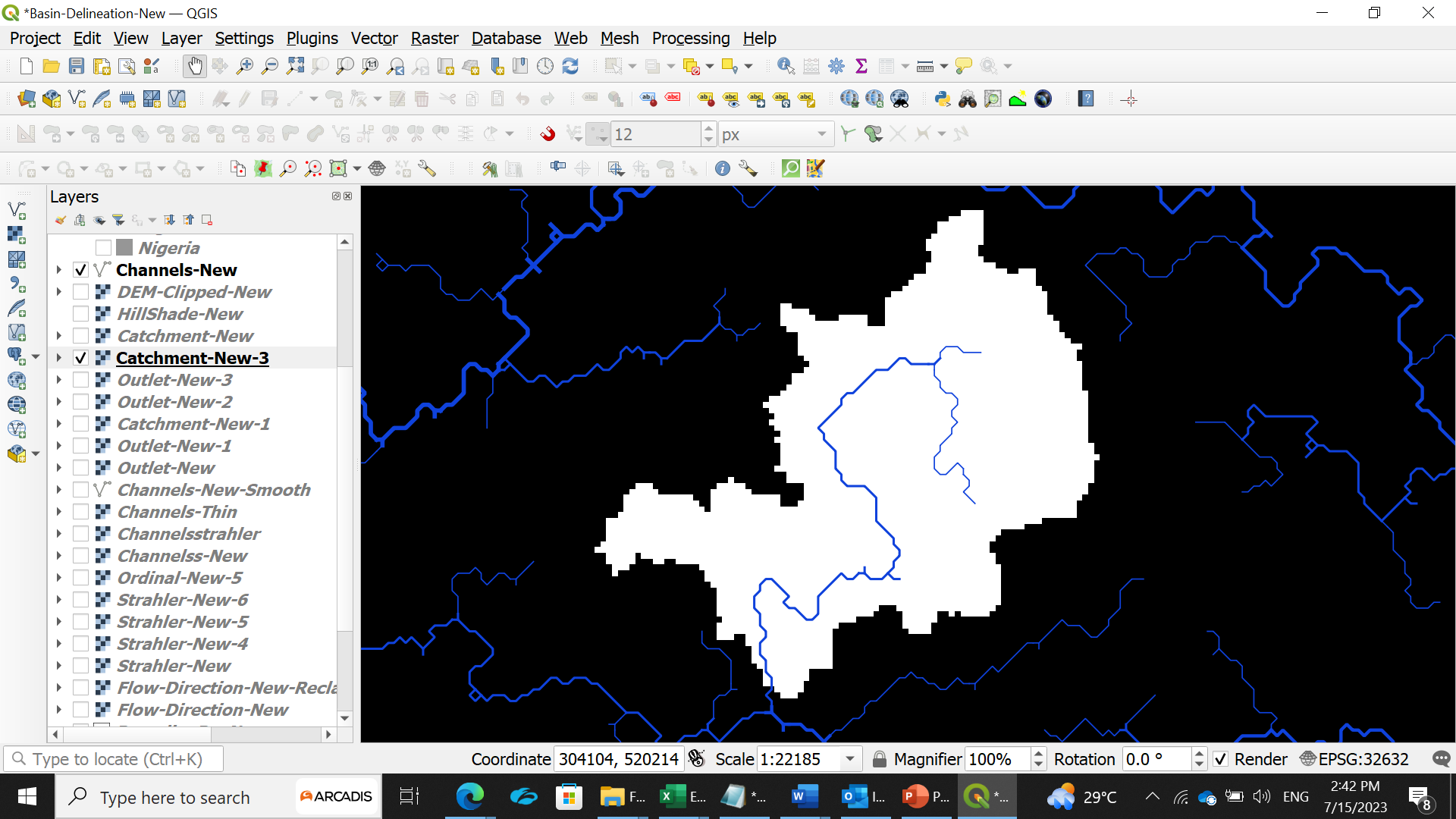


**Fig. S5** Delineated sub-catchment with river channels in raster format. Derived using channel network and drainage basins algorithm available in QGIS 3.4.8 processing modules library


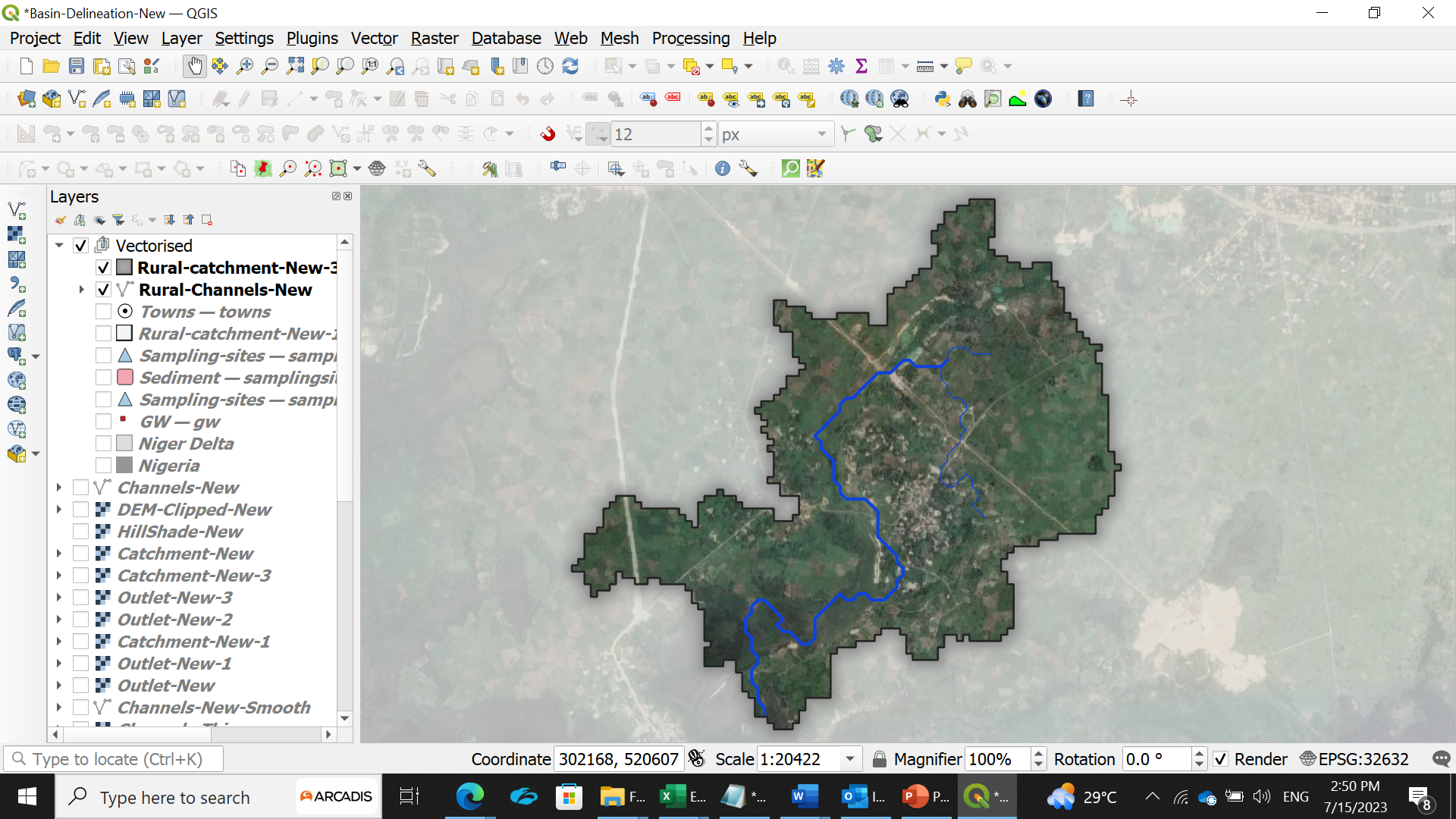


**Fig. S6** Delineated sub-catchment with its main river channel in polygonised format. Derived using the raster to vector conversion tool available in QGIS 3.4.8 raster tool bar


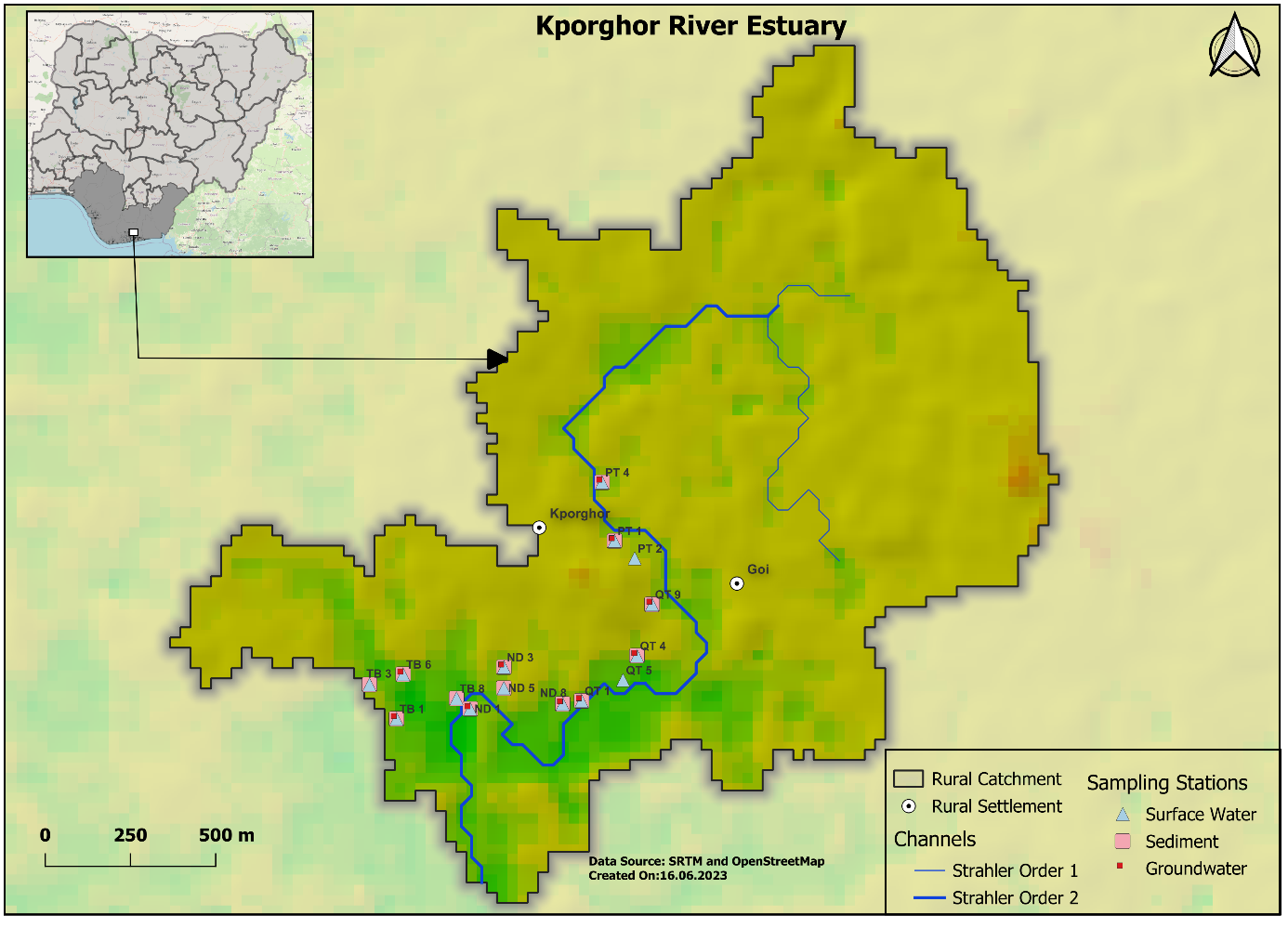
**Fig. S7**.Styling the delineated sub-catchment

**Fig. S8**. RQ_(NCs)_ and RQ_(MPCs)_ of total PAHs in Kporghor River sediments.

| 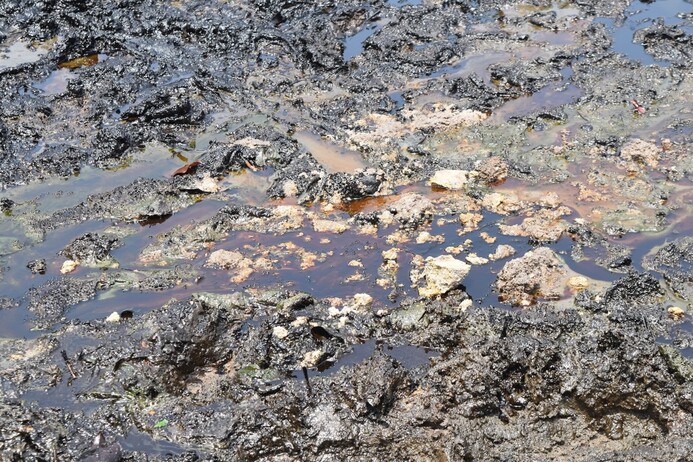  **(a)** 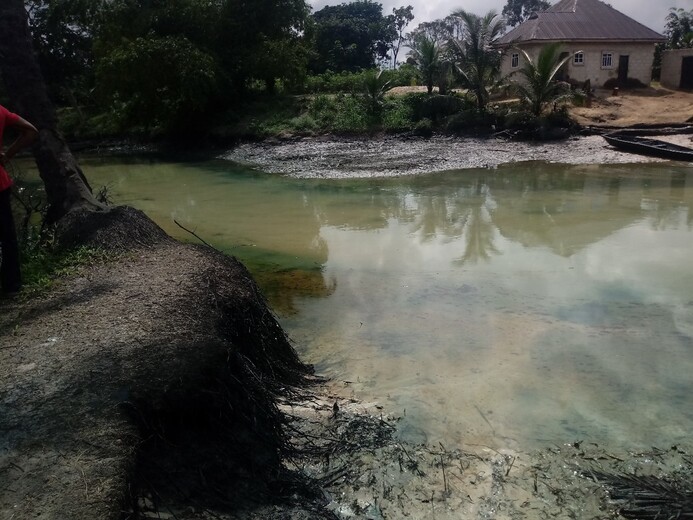 | 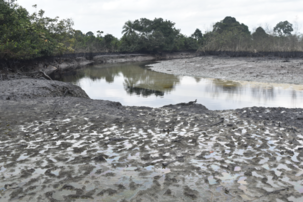  **(b)**  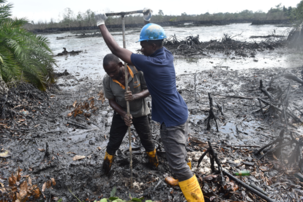 |
| --- | --- |
| **(c)** | **(d)** |

**Fig.S9** Photographs taken during field sampling: (a) Oil-coated sediment revealed at low tide mark, (b) Petroleum oil film on the main river channel surface during out-going tide, (c) Shoreline covered in tar and petroleum oil film on main river channel surface, and (d) Petroleum-coated main river channel bed exposed at low tide mark with impacted large mangrove area in the background.

# Sediment, Surface- and groundwater TPH and PAH concentrations in Kporghor River Estuary

**Table S1** Summary statistics of TPH and PAHs concentrations in Kporghor River Estuary surface and sub-surface sediments

| Site ID | Coordinates | | TPH (mg/kg) | | PAHs (mg/kg) | |
| --- | --- | --- | --- | --- | --- | --- |
|  | Latitude | Longitude | Surface | Sub-surface | Surface | Sub-surface |
| TB1 | 4.699639 | 7.226278 | 15900 | 34.7 | 4.95 | <0.01 |
| TB3 | 4.700500 | 7.226056 | 129 | 32.3 | <0.01 | <0.01 |
| TB6 | 4.700778 | 7.226944 | 20600 | 101 | 5.69 | <0.01 |
| TB7 | 4.700528 | 7.227806 | 968 | 985 | 9.55 | <0.01 |
| TB8 | 4.700139 | 7.228361 | 14700 | 65.8 | 4.28 | <0.01 |
| ND1 | 4.699861 | 7.228722 | 516 | 15.5 | 0.07 | <0.01 |
| ND3 | 4.700972 | 7.229611 | 1050 | 174 | 0.34 | 0.09 |
| ND5 | 4.700417 | 7.229611 | 13200 | 729 | 1.99 | 0.46 |
| ND8 | 4.700000 | 7.231167 | 9500 | 354 | 6.29 | 0.12 |
| QT1 | 4.700083 | 7.231667 | 2020 | 43.7 | 0.32 | 0.01 |
| QT4 | 4.700778 | 7.232806 | 7420 | 35. | 2.93 | <0.01 |
| QT9 | 4.702650 | 7.233524 | 13700 | 37.1 | 5.71 | <0.01 |
| PT1 | 4.704333 | 7.232528 | 210 | 77.4 | <0.01 | 0.02 |
| PT4 | 4.705889 | 7.232194 | 2080 | 41.4 | 0.47 | <0.01 |
| Total(mg/kg) |  |  | 102000 | 2730 | 42.6 | 0.70 |
| Mean(mg/kg) |  |  | 7290 | 195 | 3.55 | 0.14 |
| Range(mg/kg) |  |  | 129 – 20600 | 15.5 - 985 | 0.07 - 9.55 | 0.01 -0.46 |

**Table S2** Individual PAHs concentrations and frequency of detection (FD) in Kporghor River Estuary surface and sub-surface sediments

| PAHs | Surface Sediment | | | Sub-surface Sediment | | |
| --- | --- | --- | --- | --- | --- | --- |
|  | Range  (mg/kg) | Mean  (mg/kg) | FD (%) | Range  (mg/kg) | Mean  (mg/kg) | FD (%) |
| Naphthalene | <0.01–0.46 | 0.16 | 64.3 | <0.01-0.03 | 0.03 | 7.14 |
| Acenaphthylene | <0.01–1.22 | 0.21 | 85.7 | <0.01-0.03 | 0.02 | 21.4 |
| Acenaphthene | <0.01-0.68 | 0.18 | 85.7 | <0.01-0.02 | 0.01 | 21.4 |
| Fluorene | <0.01-0.45 | 0.12 | 78.6 | <0.01-0.02 | 0.01 | 21.4 |
| Anthracene | <0.01-1.89 | 0.77 | 92.9 | <0.01-0.07 | 0.07 | 21.57 |
| Phenanthrene | <0.01-1.81 | 0.80 | 92.9 | <0.01-0.08 | 0.09 | 21.57 |
| Fluoranthene | <0.01-0.63 | 0.16 | 78.6 | <0.01-0.02 | 0.02 | 14.28 |
| Pyrene | <0.01-0.64 | 0.18 | 85.7 | <0.01-0.01 | 0.01 | 14.28 |
| Benzo(a)anthracene | <0.01-2.84 | 0.37 | 78.6 | <0.01-0.07 | 0.07 | 7.14 |
| Chrysene | <0.01-0.49 | 0.15 | 64.3 | <0.01-0.13 | 0.13 | 7.14 |
| Benzo(b)fluoranthene | <0.01-0.43 | 0.16 | 71.4 | <0.01-<0.01 | - | - |
| Benzo(k)fluoranthene | <0.01-0.53 | 0.11 | 71.4 | <0.01-<0.01 | - | - |
| Benzo(a)pyrene | <0.01-0.34 | 0.15 | 71.4 | <0.01-<0.01 | - | - |
| Dibenzo(a,h)anthracene | <0.01-0.12 | 0.08 | 57.1 | <0.01-<0.01 | - | - |
| Indeno(1,2,3-cd) pyrene | <0.01-0.1 | 0.05 | 57.1 | <0.01-<0.01 | - | - |
| Benzo(g,h,i)perylene | <0.01-0.13 | 0.06 | 42.9 | <0.01-<0.01 | - | - |
| ΣPAHs | <0.01-9.55 | 3.55 |  | <0.01-0.46 | 0.46 |  |
| ΣLMW | <0.01-4.56 | 1.98 |  | <0.01-0.25 | 0.04 |  |
| ΣHMW | <0.01-4.99 | 1.07 |  | <0.01-0.21 | 0.07 |  |

^Sum of 16 US EPA priority PAHs (ΣPAHs), Sum of low molecular weight(ΣLMW), Sum of high molecular weight(ΣHMW)^

**Tab1e S3**: RQ_(NCs)_ and RQ∑PAHs_(NCs)_ values for sediments from the Kporhor River Estuary.

| Stations | Nap | Acy | Ace | Flu | Phe | Ant | Flua | Pyr | BaA | Chr | BpF | BkF | BaP | Inp | DahA | BghiP | ∑PAHs |
| --- | --- | --- | --- | --- | --- | --- | --- | --- | --- | --- | --- | --- | --- | --- | --- | --- | --- |
| TB 1 | 71.429 | 7.500 | 108.333 | 375.000 | 354.902 | 1441.667 | 1.538 | 33.333 | 63.889 | 1.776 | 5.556 | 0.417 | 0.370 | 1.481 | 0.169 | 0.667 | 13.351 |
| TB 3 | 7.143 | 0.000 | 0.000 | 0.000 | 0.000 | 0.000 | 0.000 | 0.000 | 0.000 | 0.000 | 0.000 | 0.000 | 0.000 | 0.000 | 0.000 | 0.000 | 0.027 |
| TB 6 | 214.286 | 83.333 | 183.333 | 8.333 | 313.725 | 1575.000 | 21.154 | 25.000 | 55.556 | 1.589 | 25.000 | 2.500 | 12.593 | 1.111 | 1.017 | 0.533 | 15.602 |
| TB 8 | 78.571 | 291.667 | 175.000 | 8.333 | 219.608 | 941.667 | 0.385 | 250.000 | 55.556 | 1.495 | 91.667 | 9.167 | 0.370 | 1.481 | 0.847 | 0.400 | 11.736 |
| ND 1 | 0.000 | 8.333 | 8.333 | 8.333 | 1.961 | 8.333 | 0.769 | 0.000 | 0.000 | 0.000 | 0.000 | 0.000 | 0.000 | 0.000 | 0.000 | 0.000 | 0.192 |
| ND 3 | 21.429 | 33.333 | 25.000 | 8.333 | 9.804 | 33.333 | 1.154 | 33.333 | 11.111 | 0.093 | 0.000 | 0.000 | 0.741 | 0.000 | 0.000 | 0.000 | 0.932 |
| ND 5 | 0.000 | 16.667 | 208.333 | 0.000 | 76.471 | 225.000 | 0.000 | 208.333 | 27.778 | 0.748 | 100.000 | 2.917 | 4.074 | 0.370 | 1.356 | 0.000 | 5.457 |
| ND 8 | 150.000 | 175.000 | 100.000 | 16.667 | 325.490 | 1450.000 | 24.231 | 533.333 | 30.556 | 0.654 | 119.444 | 0.417 | 8.519 | 1.852 | 2.034 | 0.533 | 17.247 |
| QT 1 | 0.000 | 25.000 | 8.333 | 16.667 | 3.922 | 8.333 | 1.154 | 16.667 | 19.444 | 0.000 | 2.778 | 0.417 | 3.333 | 0.000 | 0.000 | 0.000 | 0.877 |
| QT 4 | 71.429 | 191.667 | 216.667 | 341.667 | 137.255 | 616.667 | 4.231 | 50.000 | 5.556 | 0.187 | 2.778 | 3.750 | 0.000 | 3.333 | 0.678 | 0.667 | 8.034 |
| QT 9 | 57.143 | 141.667 | 158.333 | 16.667 | 347.059 | 1450.000 | 1.923 | 433.333 | 63.889 | 1.776 | 38.889 | 3.750 | 12.222 | 3.704 | 1.525 | 0.000 | 15.657 |
| PT 1 | 0.000 | 0.000 | 0.000 | 0.000 | 0.000 | 0.000 | 0.000 | 0.000 | 0.000 | 0.000 | 0.000 | 0.000 | 0.000 | 0.000 | 0.000 | 0.000 | 0.000 |
| PT 4 | 0.000 | 16.667 | 8.333 | 16.667 | 5.882 | 33.333 | 2.308 | 66.667 | 22.222 | 0.000 | 5.556 | 1.250 | 2.963 | 0.000 | 0.000 | 0.000 | 1.289 |
| TB 7 | 328.571 | 1016.667 | 566.667 | 316.667 | 131.373 | 958.333 | 6.923 | 150.000 | 788.889 | 4.579 | 41.667 | 22.083 | 10.741 | 3.704 | 1.695 | 1.733 | 26.186 |

**Table S4**: RQ_(MPCs)_ and RQ∑PAHs_(MPCs)_ values for sediments from the Kporhor River Estuary.

| Stations | Nap | Acy | Ace | Flu | Phe | Ant | Flua | Pyr | BaA | Chr | BpF | BkF | BaP | Inp | DahA | BghiP | ∑PAHs |
| --- | --- | --- | --- | --- | --- | --- | --- | --- | --- | --- | --- | --- | --- | --- | --- | --- | --- |
| TB 1 | 0.714 | 0.075 | 1.083 | 3.750 | 3.549 | 14.417 | 0.015 | 0.333 | 0.639 | 0.018 | 0.056 | 0.004 | 0.004 | 0.015 | 0.002 | 0.007 | 0.134 |
| TB 3 | 0.071 | 0.000 | 0.000 | 0.000 | 0.000 | 0.000 | 0.000 | 0.000 | 0.000 | 0.000 | 0.000 | 0.000 | 0.000 | 0.000 | 0.000 | 0.000 | 0.000 |
| TB 6 | 2.143 | 0.833 | 1.833 | 0.083 | 3.137 | 15.750 | 0.212 | 0.250 | 0.556 | 0.016 | 0.250 | 0.025 | 0.126 | 0.011 | 0.010 | 0.005 | 0.156 |
| TB 8 | 0.786 | 2.917 | 1.750 | 0.083 | 2.196 | 9.417 | 0.004 | 2.500 | 0.556 | 0.015 | 0.917 | 0.092 | 0.004 | 0.015 | 0.008 | 0.004 | 0.117 |
| ND 1 | 0.000 | 0.083 | 0.083 | 0.083 | 0.020 | 0.083 | 0.008 | 0.000 | 0.000 | 0.000 | 0.000 | 0.000 | 0.000 | 0.000 | 0.000 | 0.000 | 0.002 |
| ND 3 | 0.214 | 0.333 | 0.250 | 0.083 | 0.098 | 0.333 | 0.012 | 0.333 | 0.111 | 0.001 | 0.000 | 0.000 | 0.007 | 0.000 | 0.000 | 0.000 | 0.055 |
| ND 5 | 0.000 | 0.167 | 2.083 | 0.000 | 0.765 | 2.250 | 0.000 | 2.083 | 0.278 | 0.007 | 1.000 | 0.029 | 0.041 | 0.004 | 0.014 | 0.000 | 0.055 |
| ND 8 | 1.500 | 1.750 | 1.000 | 0.167 | 3.255 | 14.500 | 0.242 | 5.333 | 0.306 | 0.007 | 1.194 | 0.004 | 0.085 | 0.019 | 0.020 | 0.005 | 0.172 |
| QT 1 | 0.000 | 0.250 | 0.083 | 0.167 | 0.039 | 0.083 | 0.012 | 0.167 | 0.194 | 0.000 | 0.028 | 0.004 | 0.033 | 0.000 | 0.000 | 0.000 | 0.009 |
| QT 4 | 0.714 | 1.917 | 2.167 | 3.417 | 1.373 | 6.167 | 0.042 | 0.500 | 0.056 | 0.002 | 0.028 | 0.038 | 0.000 | 0.033 | 0.007 | 0.007 | 0.080 |
| QT 9 | 0.571 | 1.417 | 1.583 | 0.167 | 3.471 | 14.500 | 0.019 | 4.333 | 0.639 | 0.018 | 0.389 | 0.038 | 0.122 | 0.037 | 0.015 | 0.000 | 0.157 |
| PT 1 | 0.000 | 0.000 | 0.000 | 0.000 | 0.000 | 0.000 | 0.000 | 0.000 | 0.000 | 0.000 | 0.000 | 0.000 | 0.000 | 0.000 | 0.000 | 0.000 | 0.000 |
| PT 4 | 0.000 | 0.167 | 0.083 | 0.167 | 0.059 | 0.333 | 0.023 | 0.667 | 0.222 | 0.000 | 0.056 | 0.013 | 0.030 | 0.000 | 0.000 | 0.000 | 0.013 |
| TB 7 | 3.286 | 10.167 | 5.667 | 3.167 | 1.314 | 9.583 | 0.069 | 1.500 | 7.889 | 0.046 | 0.417 | 0.221 | 0.107 | 0.037 | 0.017 | 0.017 | 0.262 |

**Table S5:** Petroleum levels in surface and ground water samples in Kporghor River Estuary

| Site ID | Coordinates | | TPH (mg/L) | | | PAH (mg/L) | | |
| --- | --- | --- | --- | --- | --- | --- | --- | --- |
|  | Latitude | Longitude | | Surface water | Groundwater | | Surface water | Groundwater |
| TB 1 | 4.699639 | 7.226278 | | 156 | 3.18 | | 0.66 | <0.01 |
| TB 3 | 4.700500 | 7.226056 | | 485 | nd | | 1.09 | nd |
| TB 6 | 4.700778 | 7.226944 | | 141 | 350 | | 0.83 | 0.12 |
| TB 8 | 4.700139 | 7.228361 | | 397 | nd | | 0.41 | nd |
| ND 1 | 4.699861 | 7.228722 | | 404 | 10.3 | | 0.56 | <0.01 |
| ND 3 | 4.700972 | 7.229611 | | 51.9 | 473 | | 0.08 | 0.28 |
| ND 5 | 4.700417 | 7.229611 | | 19.8 | nd | | 0.02 | nd |
| ND 8 | 4.700000 | 7.231167 | | 50.7 | 306 | | 0.11 | 0.09 |
| QT 1 | 4.700083 | 7.231667 | | 19.4 | 52.3 | | 0.01 | 0.02 |
| QT 4 | 4.700778 | 7.232806 | | 44.1 | 7.88 | | 0.26 | <0.01 |
| QT 5 | 4.700638 | 7.232762 | | 10.0 | nd | | <0.01 | nd |
| QT 9 | 4.702650 | 7.233524 | | 620 | 21.3 | | 0.40 | 0.02 |
| PT 1 | 4.704333 | 7.232528 | | 171 | 143 | | 0.64 | 0.08 |
| PT 2 | 4.704384 | 7.232638 | | 244 | nd | | 0.10 | nd |
| PT 4 | 4.705889 | 7.232194 | | 268 | 31.6 | | 0.15 | <0.01 |
| Total |  |  | | 3080 | 1400 | | 5.32 | 0.61 |
| Mean (mg/L) |  |  | | 205 | 140 | | 0.35 | 0.06 |
| Range (mg/L) |  |  | | 10.0 – 620 | 3.18 – 473 | | <0.01 – 1.09 | <0.01 – 0.28 |

^nd represents not determined^

**Table S6** Individual PAHs concentrations and frequency of detection (FD) in Kporghor River Estuary surface water and groundwater samples

| PAHs | Surface water | | | Groundwater | | |
| --- | --- | --- | --- | --- | --- | --- |
|  | Range (mg/L) | Mean (mg/L) | FD (%) | Range | Mean | FD (%) |
| Naphthalene | <0.01-0.2 | 0.03 | 60.0 | <0.01 -0.01 | 0.002 | 20 |
| Acenaphthylene | <0.01-0.15 | 0.03 | 53.3 | <0.01 -0.01 | 0.002 | 20 |
| Acenaphthene | <0.01-0.15 | 0.04 | 73.3 | <0.01 -0.01 | 0.004 | 40 |
| Fluorene | <0.01- 0.08 | 0.01 | 40.0 | <0.01 -0.04 | 0.008 | 40 |
| Anthracene | <0.01 – 0.23 | 0.08 | 93.3 | <0.01 -0.07 | 0.017 | 60 |
| Phenanthrene | <0.01 – 0.24 | 0.086 | 86.7 | <0.01 -0.11 | 0.021 | 60 |
| Fluoranthene | <0.01- 0.06 | 0.02 | 60.0 | <0.01 -0.01 | 0.001 | 10 |
| Pyrene | <0.01- 0.06 | 0.02 | 60.0 | <0.01 -0.03 | 0.004 | 20 |
| Benzo(a)anthracene | <0.01- 0.03 | 0.01 | 26.7 | <0.01 -0.00 | 0.000 | 0 |
| Chrysene | <0.01- 0.03 | 0.01 | 13.3 | <0.01 -0.00 | 0.000 | 0 |
| Benzo(b)fluoranthene | <0.01- 0.04 | 0.02 | 53.3 | <0.01 -0.01 | 0.002 | 20 |
| Benzo(k)fluoranthene | <0.01- 0.03 | 0.01 | 73.3 | <0.01 -0.00 | 0.000 | 0 |
| Benzo(a)pyrene | <0.01- 0.02 | 0.01 | 20.0 | <0.01 -0.00 | 0.000 | 0 |
| Dibenzo(a,h)anthracene | <0.01- 0.04 | 0.01 | 46.7 | <0.01 -0.00 | 0.000 | 0 |
| Indeno(1,2,3-cd) pyrene | ND | ND | 0.00 | <0.01 -0.00 | 0.000 | 0 |
| Benzo(g,h,i)perylene | <0.01- 0.01 | 0,01 | 6.67 | <0.01 -0.00 | 0.000 | 0 |
| ΣPAHs | <0.01-1.09 | 0.38 |  | <0.01 – 0.28 | 0.06 |  |
| ΣLMW | 0.01-1.01 | 0.29 |  | <0.01 – 0.24 | 0.05 |  |
| ΣHMW | 0.00-0.24 | 0.82 |  | <0.01 – 0.04 | 0.01 |  |

^Sum of 16 US EPA priority PAHs (ΣPAHs), Sum of low molecular weight (ΣLMW), Sum of high molecular weight(ΣHMW).^
